# Tumor Microenvironment Modulation via Gold Nanoparticles Targeting Malicious Exosomes: Implications for Cancer Diagnostics and Therapy

**DOI:** 10.3390/ijms18010162

**Published:** 2017-01-14

**Authors:** Catarina Roma-Rodrigues, Luís R. Raposo, Rita Cabral, Fabiana Paradinha, Pedro V. Baptista, Alexandra R. Fernandes

**Affiliations:** UCIBIO, Departamento de Ciências da Vida, UCIBIO Faculdade de Ciências e Tecnologia, Universidade Nova de Lisboa, 2829-516 Caparica, Portugal; catromar@fct.unl.pt (C.R.-R.); luismrraposo@gmail.com (L.R.R.); ritamccabral@gmail.com (R.C.); f.paradinha@campus.fct.unl.pt (F.P.)

**Keywords:** exosomes, tumor microenvironment, gold nanoparticles, cancer diagnostics, cancer therapy

## Abstract

Exosomes are nanovesicles formed in the endosomal pathway with an important role in paracrine and autocrine cell communication. Exosomes secreted by cancer cells, malicious exosomes, have important roles in tumor microenvironment maturation and cancer progression. The knowledge of the role of exosomes in tumorigenesis prompted a new era in cancer diagnostics and therapy, taking advantage of the use of circulating exosomes as tumor biomarkers due to their stability in body fluids and targeting malignant exosomes’ release and/or uptake to inhibit or delay tumor development. In recent years, nanotechnology has paved the way for the development of a plethora of new diagnostic and therapeutic platforms, fostering theranostics. The unique physical and chemical properties of gold nanoparticles (AuNPs) make them suitable vehicles to pursuit this goal. AuNPs’ properties such as ease of synthesis with the desired shape and size, high surface:volume ratio, and the possibility of engineering their surface as desired, potentiate AuNPs’ role in nanotheranostics, allowing the use of the same formulation for exosome detection and restraining the effect of malicious exosomes in cancer progression.

## 1. Introduction

Exosomes are vesicles ranging in size from 30 to 100 nm with an important role in cell communication, being preeminent for tumor microenvironment maturation and cancer progression [1]. Despite their small size, exosomes have a complex structure composed of a lipid bilayer containing membrane proteins that surround lumen-containing proteins, mRNAs, and microRNAs (miRNAs). ExoCarta, an exosome database, highlights the contents identified in exosomes in multiple organisms [2]: more than 41,800 proteins, 4900 mRNAs, and 2800 miRNAs [3], with locations in several subcellular compartments [4]. The specific composition of exosomes seems to depend on the cell type or tissue and may differ by physiological condition [5]. For instance, exosomes derived from antigen-presenting cells present at their surface the major histocompatibility complex (MHC) I and II, whereas exosomes derived from oligodendrocytes contain myelin proteins [6,7].

Additional remarkable features of exosomes are: (i) their composition varies according to the cell of origin and process of biogenesis, with cancer-cell-derived exosomes generally reflecting the tumor stage of the cell of origin [8,9,10]; (ii) exosomes are stable in circulation, being found in body fluids, including blood, saliva, breast milk, and urine, which indicates that circulating exosomes may be suitable biomarkers for cancer diagnosis and prognosis [11]; and (iii) they are able to alter the phenotype of a recipient cell, being responsible for the tumor microenvironment maturation and cancer progression [12,13]. Understanding the role of exosomes in tumorigenesis prompted a new era in cancer diagnosis and therapy, taking advantage of the use of circulating exosomes as tumor biomarkers (reviewed in [14,15]) and targeting cancer-cell-derived exosomes (herein named *malicious exosomes*) to inhibit or delay tumor development [16].

In recent years, nanotechnology has paved the way for the development of a plethora of new diagnostic and therapeutic platforms, fostering theranostics. Among these nanoplatforms, nucleic acid nanocarriers constitute a promising approach for the efficient delivery of antisense oligonucleotides [17]. Examples of these nanocarriers are liposomes, polymeric nanoparticles, viral vectors, and most recently gold nanoparticles [18]. Gold nanoparticles (AuNPs) exhibit unique physical and chemical properties, which turns them into a powerful tool for imaging, diagnosis, and therapy, with fewer side effects [19,20]. The fact that they can be modulated in shape, size, and composition, together with their size (1–100 nm), high surface:volume ratio, and the possibility of engineering their surface as desired, mean nanoparticles can be actively targeted to specific cells to modulate the circulation time in the organism (reviewed in [21,22,23]). Because nanometer-size particles are sufficiently large to contain different targeting moieties and a variety of drug molecules, they may allow development of new strategies for therapy [20,24]. Easily synthetized, via the citrate reduction method [25], gold nanoparticles combined with biomolecules have been widely studied, with great potential for medical theranostics [26].

Here we shall address novel strategies that can be used to target malicious exosomes using gold nanoparticles as vectorization platforms. Starting from the mechanisms involved in exosomes biogenesis and uptake by secondary cells, the relevance of exosomes in the modulation of tumor microenvironment and their role in cancer diagnostics and therapy, we shall then describe the potential of AuNPs for cancer nanotheranostics, highlighting exosome-based targets and strategies to accomplish tumor growth inhibition.

## 2. Exosomes’ Biogenesis

Exosomes are formed in endosomal pathway after the fusion of multivesicular bodies (MVBs) with the plasma membrane [8,27]. However, the exact mechanisms involved in exosomes’ formation and cargo sorting are not completely understood.

### 2.1. Multivesicular Budding

**Figure 1:**
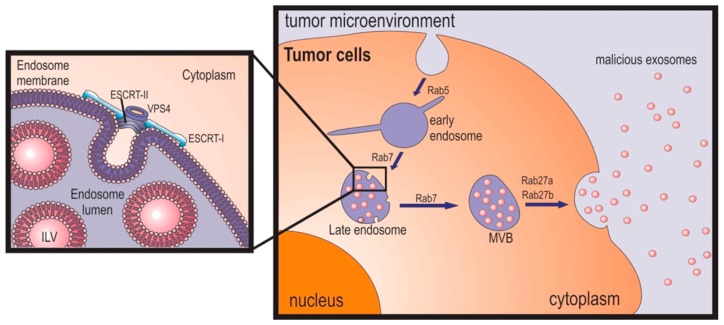
Formation and release of malicious exosomes. Exosomes are formed in the endosomal pathway. Early exosomes’ maturation occurs while they migrate from the cell periphery towards the nucleus by the formation of intraluminal vesicles (ILV) in a process mediated by endosomal sorting complexes required for transport (ESCRT) and auxiliary proteins (left image) [32]. Late endosomes, or multivesicular bodies (MVB), migrate to the periphery and ultimately will fuse with the membrane releasing the intraluminal vesicles (ILVs), which are then called exosomes. The migration process of the endosomes is mediated by different proteins belonging to the Rab GTPases family.

Exosomes are formed in the endosomal pathway (Figure 1); the process starts with the engulfment of small clathrin-coated and non-clathrin-coated vesicles formed in the plasma membrane, which are immediately transported to the network of early endosomes located at the periphery of the cell [27]. Early endosomes, which display a tubular appearance, can fuse with endocytic vesicles or suffer homotypic fusion and maturation, converting into late endosomes [27,28,29]. The fusion of early endosomes with endocytic vesicles marks their content for degradation, recycling, or secretion [30,31,32]. The clathrin-coated vesicles’ fusion with early endosomes, as well as their homotypic fusion, is mediated by the Rab5 protein [33], while the transport from early to late endosomes and lysosomes is mediated by Ras-related protein Rab-7a (RAB7A) [34]. The levels of Ras-related protein Rab-5A (RAB5A) fluctuate in singular early endosomes that migrate from the plasmatic membrane into the center of the cell, where RAB5A is exchanged for RAB7A [35].

The process of early endosomes’ maturation into late endosomes involves acidification of the endosome lumen due to vacuolar ATPases (V-ATPases), changes in protein content, and inward budding of the membrane, resulting in the formation of intraluminal vesicles (ILVs) [9,29,36,37]. Accumulation of ILVs in late endosomes originates multivesicular bodies (MVBs) in a process involving the assembly of approximately 20 proteins to form the endosomal sorting complexes required for transport (ESCRT) and auxiliary proteins, such as Programmed Cell Death 6 Interacting Protein (PDCD6IP/ALIX), Vacuolar Protein Sorting-Associated Protein VTA1 Homolog (VTA1), and Vacuolar protein sorting-associated protein 4A (VPS4) (reviewed in [27,38]). It is believed that while the ESCRT-0, ESCRT-I, and ESCRT-II complexes are responsible for the recognition and sequestration of ubiquitinated proteins targeted for lysosomal degradation, the ESCRT-III complex is involved in membrane budding and scission of ILVs [32,38]. However, there is no consensus on the ESCRT subunits, since the exosome biogenesis in different cell types seems to be accomplished by the action of distinct protein members of the ESCRT complex [9]. An ESCRT-independent mechanism has also been described for exosome biogenesis, which resembles the events elicited by budding viruses, involving membrane budding sustained by lipids (e.g., lysobisphosphatidic acid (LBPA) and ceramide) and tetraspanins proteins [9,39,40,41]. A balance between ESCRT-dependent and ESCRT-independent mechanisms seems to occur within the cells [26]. Distinct multivesicular-endosomal populations coexist in the same cell, with ILVs presenting heterogeneous sizes and compositions [8,9,10].

MVBs formation seems to be stimulated by several factors, including phosphatidylinositol 3-phosphate (PIP3), growth factors (e.g., Epidermal Growth factor (EGF)), hepatocyte growth factor-regulated tyrosine kinase substrate (HRS), pro-inflammatory proteins (e.g., PDGF-BB and Tumor necrosis factor alpha (TNF-α)), ubiquitination of the cytosolic tail of endocytosed proteins, cellular membrane topology, increase of intracellular calcium, depolarization induced by potassium, and hypoxia [40,42,43,44,45]. Moreover, the exosome secretion is enhanced by the activation of tumor suppressor-activated pathway-6 (TSAP6) and CHMP4C mediated by the tumor suppressor p53, observed in stress-induced cells [46].

### 2.2. Exosome Cargo Sorting

#### 2.2.1. Protein Sorting

**Figure 2:**
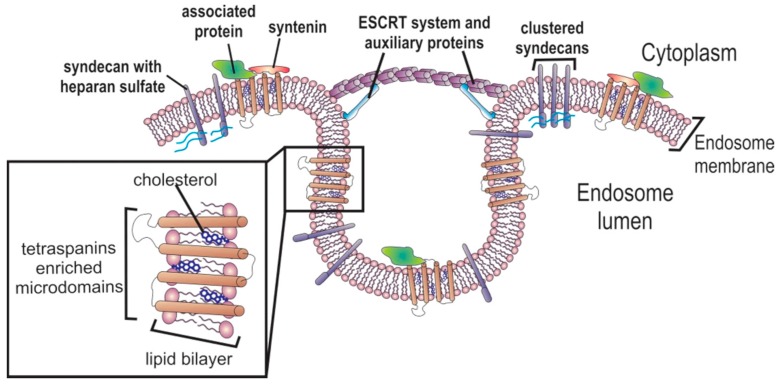
Active protein sorting into Intraluminal vesicles. During the intraluminal vesicles formation in the endosomes, proteins are sorted by the heparanase-syntenin-ALIX-ESCRT mechanism. Syndecans with long heparan sulfate chains are trimmed by heparanase and clustered after proteolytic cleavage. Syntenin couples to endosomal sorting complexes required for transport (ESCRT) machinery via Alix protein, then recruits the clustered syndecans with associated proteins and growth factors. Proteins associated with tetraspanin-enriched microdomains are probably inserted in ILVs via CD63, which is also recruited by syntenin [49].

It is mainly during ILV formation that exosome composition is defined [8,9,47]. As already mentioned above, the sorting of ubiquitinated proteins for degradation is mediated by ESCRT-0, ESCRT-I, and ESCRT-II machinery. However, protein sorting into exosomes seems to be independent of these mechanisms [48]. Two different mechanisms are likely to be involved in protein sorting into exosomes, a heparanase-syntenin-ALIX-ESCRT-dependent mechanism, and an independent mechanism [49]. In the heparanase-syntenin-ALIX-ESCRT-dependent mechanism (Figure 2), the heparanases located at the endosomal membrane cleave the long heparan sulfate chains of syndecans into shorter ones, allowing their clustering [49]. Syndecans complexes are then recruited by the cytoplasmic adaptor syntenin-1, which interacts in its turn with ESCRT-III machinery by the ALIX protein that is involved in membrane budding and protein sorting [48,49]. Different constituents of the heparanase-syntenin-ALIX-ESCRT machinery are involved in protein cargo sorting. For example, while the hepatocyte growth factor and vascular endothelial growth factor (VEGF) accumulation is stimulated by heparanase [50], recruitment of CD63 is mediated by ALIX and syntenin-1 [51]. ARF6 and PLD2 are regulators of this machinery [40]. However, other mechanisms seem to modulate protein sorting into ILVs, since CD9, CD81, and flotilin-1 proteins’ presence in exosomes is not affected by heparanase [48]. It is likely that GAIP-interacting protein C terminus (GIPC), which is involved in the trafficking of transmembrane proteins to endocytic vesicles, controls exosome biogenesis and influences exosome content [52].

Networks of tetraspanin-enriched microdomains (TEMs), consisting of platforms composed of tetraspanin proteins stabilized by palmitoylation and associated with cholesterol and gangliosides, seem to be relevant for sorting membrane proteins into ILVs [9]. It is likely that the formation of a tetraspanins web is mediated by heparanase-syntenin-ALIX-ESCRT, since tetraspanin CD63 is recruited by syntenin and heparanase [48,51]. Mobius et al. suggested that while cholesterol-rich MVBs were targeted for secretion, cholesterol-poor vesicles were destined for degradation [53]. This might reflect the increased content of tetraspanin networks in ILVs’ membranes. Tetraspanins interact with several proteins, including cytoskeleton family proteins, possibly by interactions with the ezrin-radixin-moesin (ERM) family and actin, integrins and IgSF members of adhesion receptors, proteins of the immunoglobulin superfamily, proteoglycans, signaling receptors, including protein kinase C (PKC) and G protein-coupled receptors, complement regulatory proteins, enzymes including proteases, signaling enzymes, metalloproteinases, and cadherins [9,54]. For an extended and detailed description of the role of tetraspanins in exosomes cargo selection please refer to [9,55]. In the web of tetraspanins, CD151 and Tspan8 have high relevance for tumor progression and modulation of the tumor microenvironment [56]. Interestingly, comparison between exosomes derived from highly metastatic ASML (ascites, solid, metastases, lung) cells and ASML cells with CD151/Tspan81 double knockdown, showed that these tetraspanins are preponderant for exosomal-induced stroma matrix remodeling, upregulation of cytokine expression in hematopoietic cells, and driving epithelial-to-mesenchymal transition (EMT) in non-metastatic cells [56].

#### 2.2.2. Nucleic Acid Sorting

Despite not being fully understood, increasing evidence points to nucleic acid incorporation into exosomes occurring during ILV formation via an active mechanism [30]. Additionally, evidence of a passive mechanism is supported by the fact that the content and type of RNA in exosomes generally reflects the physiological state of the cell of origin [30]. The RNA of endothelial-cell-derived exosomes reflected the induced hypoxic stress and endothelial activation of the parental cells, while exposure to high sugar concentrations had no significant alterations on the exosomal RNA content [57]. miRNAs are well-conserved small non-coding RNAs (~21–25 nucleotides) involved in the post-transcriptional regulation of gene expression [58]. miRNAs are involved in biological events, such as cell proliferation and differentiation, developmental timing, cell fate, cell reprogramming, tumorigenesis, and apoptosis, among many others [59,60]. Encoded by eukaryotic nuclear DNA, they are transcribed mostly by RNA polymerase II generating miRNA primary transcripts (pri-mRNAs), which are processed by Drosha into ~70-nucleotide hairpin precursors (pre-miRNAs). These pre-miRNAs are exported from the nucleus by exportin-5, and then cut by the endonuclease Dicer, generating short double-stranded mature RNA molecules of ~21–25 nucleotides with two-nucleotide overhangs (for a more complete review, see [61,62,63]). Typically, one of the strands (passenger strand) is degraded, and the guide strand binds to proteins of the Argonaut family (Ago) and is incorporated into the RNA-induced silencing complex (RISC), which guides it to complementary sequences in target mRNA molecules, silencing them by cleavage of the mRNA molecule, shortening of the poly A tail, and/or mRNA translational repression (for a complete review, see [64,65,66]). Interestingly, Ago2 protein also seems to be involved in miRNA sorting to exosomes, which is supported by the high export into exosomes of the Ago2-dependent miR-451 [67]. Interaction of the RISC components Ago2 and GW182 with MVBs was previously described [68], which might be a mechanism to modulate miRNA content on exosomes by the cellular levels of their targeted transcript to maintain miRNA:mRNA-target homeostasis [69].

The active RNA sorting mechanism hypothesis is supported by evidence that exosomes are enriched with mRNAs and miRNAs with specific characteristics [67,70,71,72]. There is some heterogeneity in the miRNA cargo of exosomes originating from the same cell, suggesting that some exosomes lack miRNAs [73]. A comparative analysis of the miRNA content in exosomes and B cells revealed that miRNA incorporation into exosomes might be dependent on 3′-end posttranscriptional modification [72]. Koppers-Lalic and co-workers showed an enrichment of 3′-end uridylated isoforms in exosomes, while 3′-end adenylated miRNAs were mainly found in cells, suggesting a selective distribution of miRNAs [72]. Moreover, mRNA fragments contained the 3′-untranslated regions with a 25-nt zipcode with the “CTGCC” core domain and the miR-1289 binding site located in a stem-loop structure [71,74]. Furthermore, miRNA sorting might be mediated by the recognition of GGAG and UGCA motifs by sumoylated ribonucleoprotein hnRNPA2B1 [70].

### 2.3. Exosome Release

Early and late endosomes form contact points with the endoplasmic reticulum that provide local lipid exchange and protein–protein interactions that become tighter with endosomal maturation [27]. These contact points, together with cholesterol, control the movement of endosomes to the perinuclear region mediated by dynein and Rab7-interacting lysosomal protein—RIPL [27]. Late endosomes will ultimately fuse with lysosomes for protein degradation, or with the plasma membrane, secreting the ILVs to the extracellular environment, which are then called exosomes [27]. During early to late endosome maturation, tubules form that end up forming the recycling endosomes (Figure 1) [27]. The MVBs’ intracellular traffic and fusion with plasma membrane is mediated by the action of Rab GTPases (Figure 1) and Soluble NSF-attachment protein receptors (SNAREs) [75,76]. Several Rab GTPases have been described to be involved in these processes, depending on the cell type [41]. Rab11 seems to be required for exosome secretion in K562 eritroleukemia cell line [77], while Rab35 is required for their secretion in oligodendroglial cells [78]. Additionally, Rab27a and Rab27b GTPases have been reported to be associated with the regulation of exocytosis of lysosome-related organelles [41,75]. However, it is not known if these differences are due to a specific exosome release mechanism in different cells or if the different Rab act in different steps of the exosome secretion [75]. Moreover, Riches and co-workers described that in breast cancer cells, exosome secretion is dependent on their concentration in the extracellular environment, suggesting a dynamic equilibrium between exosome release and uptake [78].

## 3. Exosomes’ Uptake by Secondary Cells

The inhibition of exosome uptake with the use of antibodies and other chemical compounds made possible the recognition of several exosome internalization mechanisms, which depend on membrane fusion, endocytosis, and protein interactions. In turn, these may induce changes to recipient cells [79,80,81].

The process of membrane fusion occurs when two distinct membranes are brought into close proximity and merge, allowing transfer of information [81,82]. Several proteins such as SNAREs, Rab proteins, SM-proteins, and several proton pumps have been described to participate in this process [83,84,85]. Exosome uptake has also been shown to be dependent on several endocytic mechanisms, such as phagocytosis, clathrin- and caveolae-dependent endocytosis, macropinocytosis, and lipid-raft-mediated endocytosis [80,81]. Actin, dynamin 2, and phosphoinositide 3-kinase (PI3K) are involved in several endocytic mechanisms [80,81]. Phagocytosis is an actin-mediated mechanism that requires the presence of receptors to specific membrane proteins [86]. This process exhibits two different patterns of internalization, depending on the phagocytic capability of the cell [87]. However, it is yet to be determined if, in these cells, this process occurs for internalization purpose or for elimination. Macropinocytosis is a clathrin-/caveolae-independent endocytic pathway like phagocytosis but does not require direct contact for the uptake. Also, this mechanism is dependent on Rac1, actin, and cholesterol—which is required for the recruitment of activated Rac1 [88,89]—and requires Na^+^/H^+^ pumps [90]. Several inhibitors of Na^+^/H^+^ pumps, such as 5-(*N*-Ethyl-*N*-isopropyl)amiloride, amiloride, and bafilomycin A, have already been proven to decrease macropinocytosis uptake [91,92]. PI3K has been shown to stimulate macropinocytosis [90].

Lipid rafts are sphingolipid- and cholesterol-enriched areas in the plasma membrane that act as organizing centers [93] and may promote clathrin-independent endocytosis of exosomes [80]. Also, exosomes carry several tetraspanins that bind to integrins in recipient cells [94]. For instance, the tetraspanin Tspan8 interacts with CD49d to promote exosome uptake [94]. Also, heparan sulfate proteoglycans and lectins have been shown to be important for exosome uptake [95,96,97,98].

## 4. Malicious Exosomes and Cancer Progression

**Figure 3:**
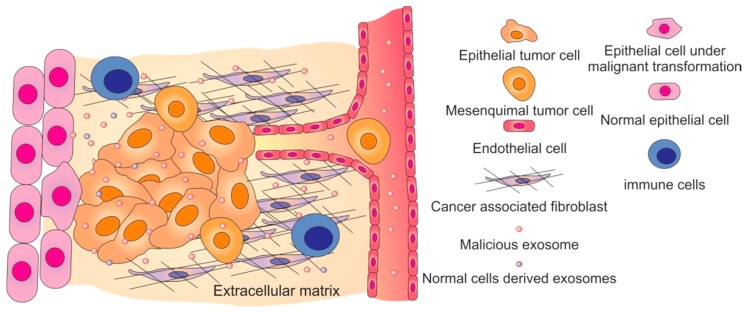
Modulation of the tumor microenvironment mediated by malicious exosomes. Malicious exosomes are involved in the maturation of the tumor microenvironment by inducing malignant transformation of normal epithelial cells, inducing the transformation of fibroblasts into cancer-associated fibroblasts, inhibiting the immune system, stimulating the angiogenic process, and inducing the epithelial to mesenchymal transition of epithelial tumor cells [1].

As discussed above, exosomes are formed in the endosomal pathway and are the result of the fusion of ILVs with early endosomes in MVBs [76]. Once released to the extracellular space, exosomes can interact with cells in the neighborhood or travel long distances, enabling the transfer of their cargo between different cells, modulating their phenotypes [43]. This modulation of a recipient cell phenotype is dependent on the origin of the exosomes: positive effects are triggered by exosomes released from normal cells, whereas negative effects arise from exosomes released from cancer cells (transfer of oncogenic features)—*Malicious exosomes* [99]. The tumor microenvironment contains multiple stromal cells, including cancer-associated fibroblasts, endothelial cells, adipocytes and infiltrating immune cells (all of which communicate with tumor cells), blood vessels, signaling molecules, and extracellular matrix (ECM) proteins (Figure 3) [59]. Paracrine communication occurring between genetically and epigenetically diverse tumor cells and the tumor microenvironment is crucial for tumor malignancy and progression (reviewed in [1,100]).

Concerning the protein cargo of malicious exosomes, proteomic analysis conducted in exosomes derived from cancer cells revealed the presence of metastatic factors (e.g., MET, S100A8, S100A9, TNC), signal transducing proteins (e.g., β-catenin, EFNB2, TNIK Wnt5B), metabolic enzymes (e.g., GAPDH, ENO1), stress response proteins (e.g., HSP90α, HSP70), cytoskeleton proteins (e.g., tubulin, actin), transporters (e.g., SLC44A2, SMVT, SLC1A5, CLIC1), proteases (e.g., PAI-1, PRSS23, CTSD, PLAU), metalloproteinases (MMPs) and their activators (e.g., MMP-14, MT1-MMP), cell surface receptors (e.g., EPHA2, TACSTD2, ROR2), miRNA metabolism proteins (e.g., Dicer), signaling glycoproteins (e.g., CD47, TSP1, and SIRPα), and even transcriptional factors (e.g., Notch, Wnt) [11,101,102,103,104,105,106]. Furthermore, exosomes are highly enriched with endosome-associated proteins (e.g., ALIX, TSG101, heat shock proteins, such as Hsp70 and Hsp90 or Rab GTPases) and plasma membrane proteins (e.g., actin, annexin, tetraspanins CD9, CD63, CD37, CD81 and CD82, integrins, and antigen-presenting molecules).

Besides mRNA and miRNA, exosomes are also composed of a diverse range of other nucleic acids, including mtDNA, piRNA, lncRNA, rRNA, snRNA, snoRNA, and tRNA [106,107]. Transmission of nucleic acids mediated by exosomes is crucial for microenvironment maturation and tumor development, as mRNA and miRNA can modulate neighbor or anatomically distant normal cells inducing tumor phenotype [1,12,13]. Even though the length of exosomal mRNA is generally no longer than 700 nucleotides, in vitro translation into full proteins was observed [108,109,110], suggesting that normal cell modulation may be attributed to mRNA transported by exosomes. However, miRNAs are also very preponderant in the genetic regulation of normal cells that end up with a malignant phenotype [58,111]. Oncogenic miRNAs, oncomirs, dysregulated in cancer cells, are known to play essential roles in cancer initiation and progression [58,66]. The influence of miRNAs in cells distant from their origin is conceivable due to their transport out of cells via exosomes, which can then enter circulation and be transported to distant sites [112]. Importantly, exosomes have been reported to protect miRNAs from degradation by RNAses [113]. Squadrito and co-workers showed that miRNA transported in macrophage-derived exosomes modulated the gene expression and biology of acceptor endothelial cells [69].

Very few studies have focused on the lipid composition of malicious exosomes [10,114]. Generally, exosomes are composed by raft-associated lipids, including cholesterol, diglycerides, sphingolipids, phospholipids, glycerophospholipids, and polyglycerophospholipids [44], and distinct lipid composition is observed between exosomes and parental cells [44,115]. Using Laser Tweezers Raman Spectroscopy, Smith and co-workers found exosomal heterogeneity in the same cell concerning the lipid content [10]. Moreover, the amounts of cholesterol and phospholipids varied between exosomes secreted by cancer and normal cells, and a role for exosomal lipids in tumor progression and drug resistance have been described [10,114]. Additionally, transport mediated by malicious exosomes of growth factors (e.g., TNF-α, EGF, and fibroblast growth factor (FGF)) is preponderant for tumor microenvironment modulation [1].

Situations of stress like exposure to hypoxia, starvation, or acidic conditions are common in the tumor microenvironment [116]. Stress conditions experienced by tumor cells promote the release and trafficking of malicious exosomes that may contribute to tumor growth and evasion since they can alter the surrounding microenvironment by modulating healthy cells, which start presenting a malignant phenotype [117]. Also, these nanosized vesicles can stimulate angiogenesis by inducing expression of vascular endothelial growth factor (VEGF) and cytokines in endothelial cells and pericytes and migration through secretion of matrix MMPs or its activators, which degrade proteins from the extracellular matrix such as collagen and fibronectin [5,99,103,116,118,119,120]. Vesicular transport via exosomes can also influence tumor-related pathways including EMT, migration, and metastasis by preparing the metastatic niche at a new anatomical location [1,100,103,121]. EMT is a conserved biological process responsible for the transition from a polarized, immotile cell, which normally interacts with the basement membrane through its basal surface, to a motile mesenchymal cell. A series of biochemical changes characterize this process, including activation of transcription factors, expression of specific cell-surface proteins, reorganization and expression of cytoskeletal proteins, production of ECM-degrading enzymes, and changes in the expression of specific miRNAs [122]. Phenotypically, the cells become less adhesive due to decreased expression of cell adhesion proteins such as E-cadherin and γ-catenin; they lose their apical–basal polarity and increase their motility and invasive potential due to the increased expression of mesenchymal markers such as vimentin, N-cadherin, fibronectin, and some matrix MMPs [65,123]. EMT culminates in the degradation of the underlying basement membrane, allowing the mesenchymal cell to migrate away from its original epithelial layer [122], which plays a crucial role in tumor invasion and metastasis, constituting an early metastatic step [66]. Moreover, exosomes serve as intercellular communication vehicles, even at a distance, and play important roles in drug resistance [100,124]. Exosomes can also be responsible for the efflux of intracellular drugs, which can be the basis of therapy resistance [112]. Stromal cells located in the tumor microenvironment secrete exosomes that modulate the invasiveness and metastatic potential of the cancer cells [1,100], and exosome release seems to be increased as the tumor progresses, with metastatic cells generally producing higher amounts of exosomes than epithelial cells [125]. Exosomes are also preponderant in drug resistance of tumor cells, since the increased secretion of exosomes containing chemotherapeutical drugs seems to be directly proportional to the drug resistance throughout the cancer cell lines [126]. Also, the mRNA, miRNA, and protein content of cancer-cell-derived exosomes seems to play a role in chemotherapy resistance [45]. As an example, docetaxel-resistant derived exosomes transporting MDR-1 confer resistance to docetaxel-sensitive cells [127,128].

Malicious exosomes play a crucial role in the modulation and shaping of the tumor microenvironment. Understanding the molecular pathways involved in exosomes’ biogenesis and recognition by normal cells is crucial for clarifying their role in tumor microenvironment modulation. Novel strategies to thwart exosome release by cancer cells or their uptake by normal cells via the effective targeting of key genes/proteins involved in these pathways have been continuously explored. Also, since exosomes are stable in circulation (detected in body fluids, including blood, saliva, breast milk, or urine), this indicates that circulating exosomes may be used as biomarkers for cancer theranostics (diagnostics and therapy) [11]. Altogether, controlling exosomes’ malicious effects may constitute a new weapon in the fight against cancer. The following sections will point out some of these strategies.

## 5. Exosomes in Cancer Diagnosis

As mentioned earlier, malicious exosomes are enriched in proteins, mRNAs, and miRNAs that are differentially expressed in cancer cells. Therefore, malicious exosomes are potential biomarkers in biological fluids, and hopefully will eliminate the need for tumor biopsies. Increased levels of circulating exosomes have been observed in the sera of patients with epithelial ovarian cancer, lung adenocarcinoma, or colorectal cancer and in the urine of patients with prostate cancer [129,130,131,132]. In ovarian cancer patients, similar levels of miR-21, miR-141, miR-200a, miR-200b, miR-200c, miR-203, miR-205, and miR-214 were found in sera exosomes and in tumor cells, revealing that it might possible to diagnose ovarian cancer using exosomes extracted from blood sera of patients instead of biopsy profiling [129]. Another report of patients with ovarian cancer demonstrated that a panel of four miRNAs (miR-373, miR-200a, miR-200b, and miR-200c) isolated from blood sera exosomes was suitable to distinguish between benign and malignant forms of tumors that correlated to shorter overall survival [133]. Analysis of the miRNA content of exosomes recovered from pleural and peritoneal effusions of patients with ovarian cancer also revealed a correlation between higher levels of miR-21, miR-23b, and miR-29a and shorter disease-free survival, and another association of higher levels of miR-21 and a shorter overall survival time [134]. Similarly, in patients with lung adenocarcinoma, the levels of miR-17, miR-21, miR-106a, miR-146, miR-155, miR-191, miR-192, miR-203, miR-205, miR-210, miR-212, and miR-214 in exosomes recovered from blood sera were similar to those encountered in tumor samples, thus making possible diagnostics in blood samples [130]. In prostate cancer patients, the presence of two well-known biomarkers in exosomes recovered from urine samples, such as the PCA-3 and TMPRSS2:ERG mRNAs, indicated an alternative strategy for early screening of the disease [135]. Also, in clear-cell renal cancer patients, higher levels of circulating miR-210 and miR-1233 in exosomes from blood sera showed a marked decrease in patients recovering from renal surgery [136]. A strong correlation of higher levels of miR-373 in circulating exosomes retrieved from the blood sera of patients with estrogen-negative, progesterone-negative, and triple-negative breast cancers was also demonstrated [137]. A set of differentially expressed miRNAs (miR-29a, miR-29b, miR-29c, miR-150, miR-155, miR-191, miR-223, miR-302d, miR-579, miR-630, miR-1246, and let-7d) positively correlate with chronic lymphocytic leukemia [138].

## 6. Exosomes in Cancer Therapy

The exploitation of exosomes for therapy, either as nanovesicles to carry therapeutics and/or devices to limit cancer progression, has been gaining momentum. One major approach consists in the inhibition of malicious exosomes’ biogenesis. However, inhibition of some pathways may be cell-dependent, which may be a limitation. As an example, while the inhibition of ceramide synthesis via the sphingomyelinase pathway reduces exosome production in myeloid-derived suppressor cells [139], the same outcomes were not observed in prostate cancer [140]. RNA-mediated silencing or knockdown of Rab27a generally results in decreased secretion of exosomes [141,142,143].

The use of exosomes in cancer therapy takes advantage of the biocompatibility, stability, and targeting ability of exosomes for targeting the delivery of proteins, RNAs, or chemotherapeutic drugs. Loading of therapeutic cargo into exosomes may be accomplished by passive or an active loading. Passive loading consists of the overexpression of RNAs, including miRNAs, shRNAs, or mRNA, in a cell culture and further collection of exosomes containing the desired cargo [144]. Exogenous loading consists of the directed introduction of the desired molecule into purified exosomes via electroporation [144]. Engineering exosomes with a therapeutic cargo and target ligands fused to extracellular proteins greatly improves the specificity and efficiency of the therapy. For example, Tian and co-workers electroporated doxorubicin into exosomes collected from immature dendritic mouse cells expressing Lamp2b fused to a tumor-targeting integrin [145]. An intravenous injection of the engineered exosomes into BALB/c nude mice with breast cancer allowed the specific target of the chemotherapeutic compound, inhibiting tumor growth [145]. Other exosome-based therapies include the removal of malicious exosomes using a hemodialysis-like procedure [146,147], or dendritic-cell-derived exosome-based vaccines [148,149,150,151]. These strategies have been put through several clinical trials but, until now, none has reached phase III [148,149,150,151,152].

## 7. Gold Nanoparticles

### 7.1. The Potential of Gold Nanoparticles

**Figure 4:**
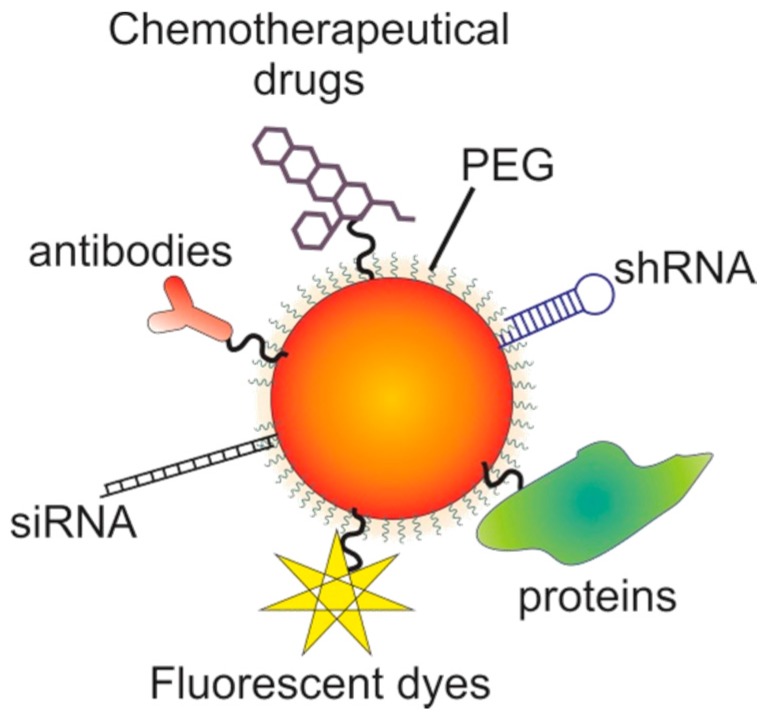
Gold nanoparticles’ (AuNPs) functionalization for theranostics. After functionalization with polyethylene glycol (PEG) for higher biocompatibility, AuNPs may be functionalized with a variety of molecules, including chemotherapeutical drugs, antibodies, small interference RNA (siRNA), short hairpin RNA (shRNA), fluorescent dyes, proteins, or a combination of several biomolecules.

AuNPs are easily synthesized in the lab in a variety of sizes and shapes, conferring them intense light absorption and scattering, high photothermal conversion rate, and photostability. Other attractive characteristics of AuNPs include their high colloidal stability, biocompatibility, and simple ligand conjugation chemistry, i.e., they may be covalently or electrostatically conjugated to a variety of biomolecules, including nucleic acids, proteins, peptides, antibodies, fluorophores, drugs, etc., conferring them targeting, therapeutic, and diagnostic capabilities [153,154,155,156]. The application of nanoparticles for cancer therapy requires stability in solutions with high protein and salt concentrations [157]. Controlling the size of the nanoparticles is important because it will influence optical and electric properties, the pharmacokinetics, biodistribution, and accumulation in the tumor site. Nanoparticles should not be smaller than 10 nm to avoid renal clearance and the surface charge must be neutral or negative to minimize nonspecific interactions with other molecules and avoid immune response [24,158]. To increase the circulation half-life of AuNPs, they are usually functionalized with biopolymers such as polyethylene glycol (PEG), which increases their hydrophilicity and therefore colloidal stability, biocompatibility, and biodistribution. The PEG layer at the AuNP surface becomes hydrated, generating an inert hydrophilic surface, which increases the AuNP stability in high salt concentrations and biological environments by preventing their aggregation. Equally, functionalization with PEG prevents the non-specific electrostatic adsorption of biomolecules, including proteins such as opsonins, which are circulating plasma proteins that mark antigens for phagocytosis. Therefore, covering AuNPs with PEG molecules prevents the recognition of AuNPs by the mononuclear phagocyte system (MPS) (which would remove AuNPs from circulation), and subsequently increases their half-life in the blood stream and biodistribution. PEG molecules can be linked to the AuNP surface via a PEG-linked thiol group (thiolated PEG), which forms a quasi-covalent bond with the AuNP surface. PEGs can be intercalated with other functional groups at the AuNP surface, or can themselves serve as linkers for the subsequent functionalization with other functional biomolecules (Figure 4). The latter requires bi-functional PEG chains, containing a thiol group at one end and an appropriate functional moiety at the other, such as an amino or carboxyl group [159], which allows a variety of biomolecules to be linked to the PEGylated AuNPs, including cell-penetrating peptides, fluorescent dyes, tumor-targeting ligands or antibodies, nucleic acids, etc.

Nucleic acid molecules modified with thiol groups at either the 3′ or 5′ ends show a strong affinity for the AuNP surface, forming quasi-covalent bonds [160]. Using this strategy, it is possible to functionalize AuNPs with single-stranded oligonucleotide (ssDNA) molecules, short interfering RNAs (siRNAs), and miRNAs [159]. In addition, AuNPs can be functionalized with a fluorophore-labeled hairpin DNA that can silence gene expression (mRNA) as well as exogenous siRNA and endogenous miRNA, while exhibiting a quantifiable fluorescence signal that is indicative of the degree of silencing [161,162,163]. In a similar way, AuNPs functionalized with ssDNA can also hybridize with complementary nucleic acid sequences in biological samples [163,164,165,166,167]. Another way to functionalize AuNPs with nucleic acids is via electrostatic interactions. Zhang et al. demonstrated that the adsorption process between nucleic acids and AuNPs is governed by electrostatic interactions, and that the charge repulsion among DNA strands and between DNA and AuNPs can be reduced with salt addition, reduction of the pH, or by using non-charged peptide nucleic acid (PNA) [168].

### 7.2. Targeting with Gold Nanoparticles

AuNPs may reach the tumor by passive or active targeting. Most solid tumors possess intrinsic characteristics, namely high vascular densities with extensive permeability and impaired lymphatic clearance, together known as the enhanced permeability and retention (EPR) effect. These characteristics originate with the process of neo-angiogenesis, which is the rapid recruitment of new blood vessels to feed the tumor as it continues to grow. Angiogenesis is triggered by the release of cytokines and other signaling molecules as the tumor needs more oxygen and nutrients, and results in an abnormal vasculature in both form and architecture, with a very disorganized endothelium presenting large fenestrations into the tumor interstitial space. These gaps, which are only present around the tumor, allow the selective penetration of nanosized molecules from the defective vasculature to the tumor site [153]. At the same time, the rapid and uncontrolled tumor growth compresses the lymphatic vessels, which end up collapsing, resulting in impaired lymph drainage. This allows the nanosized molecules to be retained at the tumor site, but not in normal tissues. Together, these processes allow the passive targeting of the tumor by the nanosized molecules. However, once the nanoconjugates reach the tumor site, they need to be internalized into the tumor cells. The extent of cellular uptake depends on factors such as the size, shape, surface charge, and lipophilicity of the AuNP conjugates. Despite this EPR effect, functionalization of AuNPs with active targeting moieties has been shown to increase the cellular uptake of nanoconjugates [169,170,171].

Typically, tumor cells overexpress a certain number of cell surface receptors (as compared with normal cells) that can be used as tumor biomarkers, i.e., markers which altered expression correlates with a specific clinical outcome or biological behavior. These cell surface receptors can be used to direct AuNPs preferentially to tumor cells. Since tumor cells will express more of these cell surface receptors than normal cells, by functionalizing AuNPs with biomolecules that bind specifically to those cell surface receptors, they will accumulate preferentially in tumor cells. To this end, AuNPs are usually functionalized with targeting ligands such as monoclonal antibodies and peptides/proteins (e.g., cell internalization peptides, transferrin, EGF), folic acid, carbohydrates, and DNA/RNA, depending on the desired cell target [21,172].

## 8. Targeting Malicious Exosomes with Gold Nanoparticles

The described properties of AuNPs make them suitable for targeting malicious exosomes and several different approaches have been proposed: (i) tackling malicious exosome biogenesis and release by AuNPs functionalized with tumor-specific targeting moieties (e.g., antibodies against overexpressed receptors) and silencing moieties that target key genes/proteins involved in exosomes biogenesis and secretion; (ii) targeting circulating malicious exosomes using AuNPs as theranostics devices via functionalization with affinity agents, including exosome-binding lectins and antibodies, aiming at exosome capture and selective retention from the entire circulatory system [146,147]; and (iii) limiting exosome uptake by secondary cells using AuNPs functionalized with inhibitors of the exosome uptake machinery (e.g., siRNAs, hairpin antisense oligonucleotides), and consequently reducing normal cells’ modification by malicious exosomes within the tumor microenvironment or at distant locations. The next sections will describe silencing and targeting strategies, either directly of vectorized by AuNPs, to modulate the way malicious exosomes modify the tumor microenvironment (summarized in Table 1).

Recent studies suggested that exomes may also mediate the transport of AuNPs [18]. Spherical nucleic acids containing a gold core were endocytosed by PC-3 prostate cancer cells, sorted into exosomes, and re-introduced into the same cell type, where they exhibit high gene knockdown [18]. In another study, the silencing capacity of the functionalized AuNPs was extended to cell lines of a different anatomical region [117]. Indeed, the exosomes secreted by breast cancer cells treated with AuNPs functionalized with an anti-*RAB27A* gene could induce *RAB27A* gene silencing in bronchial/tracheal epithelial cells [117]. The loading of AuNPs into exosomes might occur after uptake of AuNPs by endocytosis and further processing in the endosomal pathway (reviewed in [189,190]). This is supported by imaging analysis of the internalization of AuNPs by the water invertebrate *Hydra polyp* that revealed AuNPs’ internalization by cells in the highly eukaryotic conserved endosomal network [191]. These results point out to a new avenue for cancer diagnostics and therapy via gold-nanoparticle-mediated targeting of exosomes.

### 8.1. Tackling Exosome Biogenesis

It is during ILVs’ formation in MVBs that the exosome composition is determined. Hence, it is tempting to target this pathway with the intent to inhibit the secretion and manipulate the cargo of malicious exosomes. The intrinsic properties of AuNPs, including tumor passive targeting, biocompatibility, and easy functionalization, made them suitable for use as vehicles of exosome biogenesis. One approach to tackle malicious exosomes via AuNPs might be achieved by targeting MVB in tumor cells. In cancer cells, a correlation might be possible between increased exosome secretion and defective endocytosis [192]. Depletion of key subunits of the four ESCRT complexes (namely, Hrs, Tsg101, Vps22, and VPs24), resulted in larger multivesicular endosomes but a clear differentiation between early and late endosomes was still observed [173]. RNAi targeting of the auxiliary proteins Vps4A/B on MCF7 cells resulted in lower release of exosomes [51]. Overexpression of Rab5 with a point mutation that causes lower GTPase activity of the protein resulted in the impairment of early intra-endosomal trafficking and enlargement of endosomes [175]. Depletion of Rab7 by RNAi resulted in the formation of large endosomes filled with ILVs and reduced the levels of exosome release [51]. On the other hand, a decreased content of Vps4A was associated with hepatic tumor progression, being involved in the regulation of miRNA secretion in human hepatoma cells [193].

Despite not being directly related to targeting the biogenesis of exosomes, one approach that is relevant for cancer therapy takes advantage of AuNPs’ endocytosis. Using this approach, the lower pH of early endosomes when compared to the extracellular environment can be used to trigger pH-dependent drug release from the nanocarriers (reviewed in [194]).

Targeting the protein and RNA sorting machinery may allow the modulation of exosome content to reduce their malignancy. As an example, a significant decrease of exosome proteins was observed after inhibition of ARF6 mediated by RNAi [40]. The higher expression of heparanase in cancer cells is generally correlated with an increase of tumor angiogenesis, invasiveness, and metastasis [195,196,197,198]. Inhibition of the heparanase activity or disruption of the heparan sulfate structure in MCF7 cells resulted in the reduction of exosome secretion [48,51]. Indeed, it was registered that exosomes of GIPC-deficient pancreatic cancer cells had a distinct composition of the exosomes of the parental cells, also resulting in an increased sensitivity to gemcitabine, possibly by sequestering the efflux pump ABCG2 in the vesicles [52]. Considering the influence of mRNA and miRNA in tumor development, it would be expected that blocking the mRNA and miRNA internalization into exosomes would allow for constraining cancer’s progression. This might be achieved by the construction of nanoparticles targeting miRNA internalization sequences, limiting the incorporation of miRNAs containing these motifs into exosomes. Indeed, mutations in GGAG and UGCA motifs enable the modulation of miRNA cargo in exosomes [61]. Li and co-workers described a strategy based on the fluorescence-induced cleavage of a RNA molecular beacon to determine the RNA endonuclease activity of Ago2 [174]. The functionalization of nanoparticles with this beacon might ally the biological compatibility and targeting of nanoparticles with the sensitive detection of Ago2 activity described by Li and co-workers for functional studies.

The silencing of proteins involved in endosome movement into the plasmatic membrane and consequent exosome release has been demonstrated; along with knockdown of Rab27a and its effector, Slp4 (Synaptotagmin-like protein), this inhibits exosome secretion in HeLa cells, resulting in larger MVBs [142]. It was suggested that Rab27a is required for exosome docking to the plasma membrane and, when absent, vesicles will fuse with each other instead of fusing with the plasma membrane [142,199]. The secretion of exosomes can be promoted by hypoxia and the inhibition of Rab27a has been associated with reduced mobilization of neutrophils, which leads to decreased tumor growth and lung metastasis, demonstrating that Rab27a is involved in cancer progression [43,112]. Overexpression of Rab27a has been associated with the invasive and metastatic potential of human breast cancer cells by promoting the secretion of insulin-like growth factor II (IGF-II), involved in several roles in normal and breast cancer cells such as regulation of VEGF [200,201]. Gold-nanoparticle-mediated silencing of *RAB27A* in breast cancer cells resulted in a decrease of exosome secretion with no consequences for cell viability [117].

### 8.2. Tackling Circulating Exosomes

Once exosomes are released to the tumor milieu, they can act upon the paracrine communications between tumor cells and stromal cells or travel long distances in the blood or lymphatic systems [1]. The versatility of gold nanoparticles allows their use in nanotheranostics, by using the same functionalized AuNPs for diagnosis and restraining the effect of malicious exosomes in secondary cells. Malicious exosomes’ biomarkers, located at the exosome surface, including lipids, proteins, and glycoproteins, can be used to achieve this goal. Despite the high potential of the exosome-based nanotheranostics, it is still in its infancy and studies are mainly focused on AuNP-based liquid biopsies for diagnosis.

The plasmon resonance characteristics of AuNPs have been used for the design of sensors for exosome quantification in liquid biopsies [176,177,178,179]. Giuseppe and co-workers directly applied colloidal AuNPs to exosomes from multiple myeloma, monoclonal gammopathy of undetermined significance, and healthy individuals [176]. Obtained aggregation indexes determined that multiple myeloma patients produced 4-fold more exosomes than other patients [176]. In another approach, Duraichelvan and co-workers designed 3D gold nanostructures functionalized with streptavidin and biotin-Vn96 for quantification of the total exosome concentration in body fluids [177]. Oliveira-Rodriguez and co-workers designed a lateral flow immunoassay for exosome detection using CD9, CD81 tetraspanins as capture antibodies, and CD63 conjugate with gold nanoparticles for detection [178]. This test showed high sensitivity to the tetraspanin content, correlated to the concentration of exosomes and signal intensity [178]. To profile the exosome surface proteins and proteins present in exosomes lysates, Im and co-workers constructed nanohole arrays composed of holes containing AuNPs functionalized with antibodies [179]. This portable multiplexed protein analysis allowed the identification of higher expression of CD24 and EpCam in exosomes present in ascites samples from ovarian cancer patients [179].

Using another screening methodology, Angeloni and co-workers could bind rhodamine-labeled HDL-like nanoparticles to circulating exosomes containing the scavenger receptor type B-1 (SR-B1), which is a high-affinity receptor for HDL frequently found in malicious exosomes, for exosome tracking and quantification [180,181].

### 8.3. Tackling Exosome Uptake

The uptake of exosomes is dependent on the type of cell, its physiological state, and the presence of surface receptors in exosomes [30]. Targeting the lipid content of malicious exosomes may allow for creating constraints in cellular uptake and thus limiting the negative effects of exosome internalization. In the same line, blocking protein receptors in exosomes, e.g., tetraspanins and integrins highly expressed in exosomes, decreases exosome uptake by secondary cells [115]. Interestingly, Plebanek and co-workers could inhibit the cellular uptake of exosomes by binding high-density lipoprotein (HDL) nanoparticles to SR-B1 located in lipid rafts [181]. On the other hand, Paramelle and co-workers functionalized gold nanoparticles’ surface with a self-assembled monolayer of peptidol and alkane thiol ethylene glycol and then inserted the sphingolipid and cell membrane microdomain-binding peptide—SBD [182]. This functionalized AuNPs allowed the real-time visualization of the lipid rafts in the membrane of live cells [182]. The understanding of malicious exosomes’ uptake by normal cells may allow the scientific community to focus their goals on targeting and inhibiting this internalization. Several strategies have already been attempted to reduce exosome uptake by normal cells, which can be further optimized using gold nanoparticles as a vehicle. For instance, exosomes’ uptake via phagocytosis is shown to be dependent on actin cytoskeleton, PI3K, and dynamin 2. Targeting actin polymerization, PI3K and dynamin 2 would decrease exosome uptake [202,203,204,205,206]. As a fact, PIK3 inhibitors, such as wortmannin and LY294002, were used to test the necessity of functional PIK3 in exosome internalization [80]. The results showed a decrease in uptake, in a dose-dependent manner [80]. However, as phagocytosis is a crucial mechanism in fighting infections as well as in maintaining healthy tissue by removing injured cells, inhibiting this process may lead to extensive damage. Dynamin 2 is a protein of the subfamily of GTP-binding proteins encoded by the DNM2 gene, and has been demonstrated to interact with actin during vesicle formation in clathrin-mediated endocytosis (CME) [207]. It is possible to inhibit dynamin 2 activity by knockdown of the gene, which induces defects similar to those seen when PI3K was inhibited [183,184] as dynamin 2 interacts with a regulatory subunit of PI3K, stimulating dynamin’s GTPase activity [208]. In a more recent work, Yamada and co-workers reported that pharmacological inhibition of dynamin 2 decreases cell migration and filopodia formation [209]. Their findings suggest that dynamin 2 may be a possible target for cancer therapeutic. Exosome uptake by CME can also be decreased using chlorpromazine, which prevents the formation of clathrin-coated pits [185,186].

Caveolae-mediated endocytosis is a clathrin-independent internalization mechanism [210]. Caveolae are small cave-like, cholesterol-rich plasma membrane microdomains [211,212], characterized by the presence of the proteins caveolin-1 and caveolin-2 [206]. This mechanism is sensitive to cholesterol depletion agents, such as filipin, methyl-β-cyclodextrin, and simvastatin [90,91,187]. Caveolin-1 is a protein required for the formation of caveolae [91]; specific knockdown of the CAV1 gene leads to reduced caveolin-1 protein and afterwards significant impairment of uptake [188].

AuNPs functionalized with some of these targets may allow us to tackle this uptake in a fast, specific, and efficient way by taking advantage of the array of targets that can be functionalized onto a single NP (proteins, miRNAs, siRNAs, antisense hairpins, and targeting peptides).

## 9. Conclusions

As exosome relevance in tumor microenvironment evolution and maturation grows, it becomes more evident that targeting these nanovesicles is pivotal to constrain tumor microenvironment communications and tumor progression. AuNPs are great candidates for cancer therapy based on malicious exosome targeting: (i) AuNPs are naturally targeted to the tumor microenvironment through a passive mechanism; (ii) AuNPs are suitable vehicles for gene silencing of proteins involved in exosome biogenesis, limiting the increased concentration of malicious exosomes in the tumor microenvironment; (iii) plasmon resonance of AuNPs was proven a sensitive method for exosome quantification in liquid biopsies; (iv) AuNPs may be used in nanotheranostics to both quantify the malicious exosome content and inhibit malicious exosomes’ internalization by secondary cells; and (v) gene silencing mediated by AuNPs is propagated to secondary cells in an exosome-dependent way. As such, the plethora of conceptual and developed AuNP-based therapeutic systems directed at cancer cells may now be directed towards tackling exosomes, thus enhancing the efficacy of cancer treatment.

The use of AuNPs for theranostics is still in its infancy, and plenty more data on acute toxicity, bioavailability, metabolism, biological clearance, pharmacological delivery, and dose-response curves for chronic exposure are still required [153]. This task is not made easy due to the lack of standardized protocols for characterization of nanoparticle-based medicines and their biological effects. Recently, the Food and Drug Administration (FDA) and the Nanotechnology Characterization Laboratory (NCL) began to systematically draft protocols to address the safety and regulatory issues concerning these nanomedicines before clinical application [213]. Another critical issue for further translation to the clinics concerns scaling up synthesis from a research laboratory to an industrial setting, namely procedures to significantly increase production while maintaining reproducible product formulation and quality [213]. Nanotechnology is one of the major strategic objectives for research and innovation in the European Union, which defined several goals in terms of regulation, patenting, and funding the safe development and application of nanoparticle-based medicines in health and the industrial production of nanoformulations [213]. All these efforts should support this new avenue to foster cancer treatment via the targeting of malicious exosomes.

## Figures and Tables

**Table 1:** Compilation of silencing and targeting strategies used for inhibition of malicious exosomes’ biogenesis, exosomes’ uptake by secondary cells, and detection of circulating malicious exosomes.

Approach	Target	Strategy	References
*Exosome biogenesis*			
Multivesicular budding	Hrs, Tsg101, Vps22, and VPs24	Gene knockdown	[173]
	Vps4A/B	RNAi knockdown	[51]
Protein sorting	Arf6	RNAi knockdown	[40]
	Heparanase	Inhibition of activity	[48,51]
	Heparan sulfate structure	Disruption	[48,51]
miRNA sorting	GGAG and UGCA motifs	Mutations	[61]
	Ago2	RNA beacon for endonuclease activity determination	[174]
Endosome movement	Rab5	Point mutation with loss of function	[175]
	Rab7	Protein depletion	[51]
	Rab27a, Slp4	Gene knockdown	[142]
	Rab27a	AuNPs ^1^ mediated silencing	[117]
*Circulating exosomes*			
	Exosomes from multiple myeloma and MGUS ^1^	Aggregation indexes of AuNPs ^1^	[176]
	Exosomes in body fluids	3D gold nanostructures with streptavidin and biotin-Vn96	[177]
	Exosomes in body fluids	Platform with anti-CD9 and anti-CD63 to capture exosomes and AuNPs ^1^ with CD-81 for detection	[178]
	Exosomes in ascites samples from ovarian cancer patients	Nanohole arrays with AuNPs ^1^ functionalized with antibodies	[179]
	HDL ^3^	HDL-like nanoparticles with SR-B1	[180]
*Secondary cell uptake*			
Cell uptake inhibition	SR-B1 located in lipid rafts	HDL-like nanoparticles	[181]
Real-time visualization	Lipid rafts	AuNPs ^1^ with Sphingolipid binding peptide	[182]
Phagocytosis inhibition	PIk3	PIK3 inhibitors: wortmannin and LY294002	[80]
Clathrin-mediated endocytosis	Dynamin ^2^	Gene knockdown	[183,184]
	Clathrin-coated pits	Clathrin-coated pits inhibitor: chlorpromazine	[185,186]
Caveolae-mediated endocytosis	Cholesterol-rich microdomains	Cholesterol depletion agents, filipin, methyl-β-cyclodextrin, and simvastatin	[90,91,187]
	Caveolin-1	Gene knockdown	[188]

^1^ AuNPs—Gold nanoparticles; ^2^ MGUS—Monoclonal gammopathy of undetermined significance; ^3^ HDL—High Density Lipoproteins.

## Abbreviations

AuNPs	Gold nanoparticles
ECM	Extracellular matrix
EMT	Epithelial–mesenchymal transition
ESCRT	Endosomal sorting complexes required for transport
ILVs	Intraluminal vesicles
MMPs	Matrix metalloproteinases
MVBs	Multivesicular bodies
PI3K	Phosphoinositide 3-kinase

## References

[1] Roma-Rodrigues C., Fernandes A.R., Baptista P.V. (2014). Exosome in tumour microenvironment: Overview of the crosstalk between normal and cancer cells. BioMed Res. Int..

[2] Keerthikumar S., Chisanga D., Ariyaratne D., Al Saffar H., Anand S., Zhao K., Samuel M., Pathan M., Jois M., Chilamkurti N. (2016). Exocarta: A web-based compendium of exosomal cargo. J. Mol. Biol..

[3] Exocarta—Exosome Protein, RNA and Lipid Database. http://www.exocarta.org/.

[4] Burke M., Choksawangkarn W., Edwards N., Ostrand-Rosenberg S., Fenselau C. (2014). Exosomes from myeloid-derived suppressor cells carry biologically active proteins. J. Proteome Res..

[5] Hannafon B.N., Ding W.Q. (2013). Intercellular communication by exosome-derived microRNAs in cancer. Int. J. Mol. Sci..

[6] Soekmadji C., Russell P.J., Nelson C.C. (2013). Exosomes in prostate cancer: Putting together the pieces of a puzzle. Cancers.

[7] Tran T.H., Mattheolabakis G., Aldawsari H., Amiji M. (2015). Exosomes as nanocarriers for immunotherapy of cancer and inflammatory diseases. Clin. Immunol..

[8] Raposo G., Stoorvogel W. (2013). Extracellular vesicles: Exosomes, microvesicles, and friends. J. Cell Biol..

[9] Andreu Z., Yanez-Mo M. (2014). Tetraspanins in extracellular vesicle formation and function. Front. Immunol..

[10] Smith Z.J., Lee C., Rojalin T., Carney R.P., Hazari S., Knudson A., Lam K., Saari H., Ibanez E.L., Viitala T. (2015). Single exosome study reveals subpopulations distributed among cell lines with variability related to membrane content. J. Extracell. Vesicles.

[11] Simpson R.J., Lim J.W.E., Moritz R.L., Mathivanan S. (2009). Exosomes: Proteomic insights and diagnostic potential. Expert Rev. Proteom..

[12] Suetsugu A., Honma K., Saji S., Moriwaki H., Ochiya T., Hoffman R.M. (2013). Imaging exosome transfer from breast cancer cells to stroma at metastatic sites in orthotopic nude-mouse models. Adv. Drug Deliv. Rev..

[13] Taylor D.D., Gercel-Taylor C. (2013). The origin, function, and diagnostic potential of RNA within extracellular vesicles present in human biological fluids. Front. Genet..

[14] Mahmoudi K., Ezrin A., Hadjipanayis C. (2015). Small extracellular vesicles as tumor biomarkers for glioblastoma. Mol. Asp. Med..

[15] Lin J., Li J., Huang B., Liu J., Chen X., Chen X.M., Xu Y.M., Huang L.F., Wang X.Z. (2015). Exosomes: Novel biomarkers for clinical diagnosis. Sci. World J..

[16] Gernapudi R., Yao Y., Zhang Y., Wolfson B., Roy S., Duru N., Eades G., Yang P., Zhou Q. (2015). Targeting exosomes from preadipocytes inhibits preadipocyte to cancer stem cell signaling in early-stage breast cancer. Breast Cancer Res. Treat..

[17] Wang K., Kievit F.M., Zhang M. (2016). Nanoparticles for cancer gene therapy: Recent advances, challenges, and strategies. Pharmacol. Res..

[18] Alhasan A.H., Patel P.C., Choi C.H., Mirkin C.A. (2014). Exosome encased spherical nucleic acid gold nanoparticle conjugates as potent microRNA regulation agents. Small.

[19] Conde J., de la Fuente J.M., Baptista P.V. (2010). In vitro transcription and translation inhibition via DNA functionalized gold nanoparticles. Nanotechnology.

[20] Martins P., Rosa D., Fernandes A.R., Baptista P.V. (2013). Nanoparticle drug delivery systems: Recent patents and applications in nanomedicine. Recent Pat. Nanomed..

[21] Conde J., Doria G., Baptista P. (2012). Noble metal nanoparticles applications in cancer. J. Drug Deliv..

[22] Sanvicens N., Marco M.P. (2008). Multifunctional nanoparticles—Properties and prospects for their use in human medicine. Trends Biotechnol..

[23] Silva J., Fernandes A.R., Baptista P.V., Sezer A.D. (2014). Application of nanotechnology in drug delivery. Application of Nanotechnology in Drug Delivery.

[24] Heath J.R., Davis M.E. (2008). Nanotechnology and cancer. Annu. Rev. Med..

[25] Turkevich J., Stevenson P.C., Hillier J. (1951). A study of the nucleation and growth processes in the synthesis of colloidal gold. Discuss. Faraday Soc..

[26] Baptista P.V. (2012). Could gold nanoprobes be an important tool in cancer diagnostics?. Expert Rev. Mol. Diagn..

[27] Klumperman J., Raposo G. (2014). The complex ultrastructure of the endolysosomal system. Cold Spring Harb. Perspect. Biol..

[28] Keller S., Sanderson M.P., Stoeck A., Altevogt P. (2006). Exosomes: From biogenesis and secretion to biological function. Immunol. Lett..

[29] Stoorvogel W., Strous G.J., Geuze H.J., Oorschot V., Schwartz A.L. (1991). Late endosomes derive from early endosomes by maturation. Cell.

[30] Abels E.R., Breakefield X.O. (2016). Introduction to extracellular vesicles: Biogenesis, RNA cargo selection, content, release, and uptake. Cell. Mol. Neurobiol..

[31] Morelli A.E., Larregina A.T., Shufesky W.J., Sullivan M.L., Stolz D.B., Papworth G.D., Zahorchak A.F., Logar A.J., Wang Z., Watkins S.C. (2004). Endocytosis, intracellular sorting, and processing of exosomes by dendritic cells. Blood.

[32] Raiborg C., Stenmark H. (2009). The ESCRT machinery in endosomal sorting of ubiquitylated membrane proteins. Nature.

[33] Gorvel J.P., Chavrier P., Zerial M., Gruenberg J. (1991). Rab5 controls early endosome fusion in vitro. Cell.

[34] Wandinger-Ness A., Zerial M. (2014). Rab proteins and the compartmentalization of the endosomal system. Cold Spring Harb. Perspect. Biol..

[35] Rink J., Ghigo E., Kalaidzidis Y., Zerial M. (2005). Rab conversion as a mechanism of progression from early to late endosomes. Cell.

[36] Akers J.C., Gonda D., Kim R., Carter B.S., Chen C.C. (2013). Biogenesis of extracellular vesicles (EV): Exosomes, microvesicles, retrovirus-like vesicles, and apoptotic bodies. J. Neurooncol..

[37] Lafourcade C., Sobo K., Kieffer-Jaquinod S., Garin J., van der Goot F.G. (2008). Regulation of the v-ATPase along the endocytic pathway occurs through reversible subunit association and membrane localization. PLoS ONE.

[38] Babst M. (2005). A protein’s final escrt. Traffic.

[39] Trajkovic K., Hsu C., Chiantia S., Rajendran L., Wenzel D., Wieland F., Schwille P., Brugger B., Simons M. (2008). Ceramide triggers budding of exosome vesicles into multivesicular endosomes. Science.

[40] Ghossoub R., Lembo F., Rubio A., Gaillard C.B., Bouchet J., Vitale N., Slavik J., Machala M., Zimmermann P. (2014). Syntenin-ALIX exosome biogenesis and budding into multivesicular bodies are controlled by ARF6 and PLD2. Nat. Commun..

[41] Lo Cicero A., Stahl P.D., Raposo G. (2015). Extracellular vesicles shuffling intercellular messages: For good or for bad. Curr. Opin. Cell Biol..

[42] White I.J., Bailey L.M., Aghakhani M.R., Moss S.E., Futter C.E. (2006). EGF stimulates annexin 1-dependent inward vesiculation in a multivesicular endosome subpopulation. EMBO J..

[43] Kahlert C., Kalluri R. (2013). Exosomes in tumor microenvironment influence cancer progression and metastasis. J. Mol. Med..

[44] Record M., Carayon K., Poirot M., Silvente-Poirot S. (2014). Exosomes as new vesicular lipid transporters involved in cell-cell communication and various pathophysiologies. Biochim. Biophys. Acta.

[45] Yu S., Cao H., Shen B., Feng J. (2015). Tumor-derived exosomes in cancer progression and treatment failure. Oncotarget.

[46] Yu X., Harris S.L., Levine A.J. (2006). The regulation of exosome secretion: A novel function of the p53 protein. Cancer Res..

[47] Vlassov A.V., Magdaleno S., Setterquist R., Conrad R. (2012). Exosomes: Current knowledge of their composition, biological functions, and diagnostic and therapeutic potentials. Biochim. Biophys. Acta.

[48] Roucourt B., Meeussen S., Bao J., Zimmermann P., David G. (2015). Heparanase activates the syndecan-syntenin-ALIX exosome pathway. Cell Res..

[49] Stoorvogel W. (2015). Resolving sorting mechanisms into exosomes. Cell Res..

[50] Thompson C.A., Purushothaman A., Ramani V.C., Vlodavsky I., Sanderson R.D. (2013). Heparanase regulates secretion, composition, and function of tumor cell-derived exosomes. J. Biol. Chem..

[51] Baietti M.F., Zhang Z., Mortier E., Melchior A., Degeest G., Geeraerts A., Ivarsson Y., Depoortere F., Coomans C., Vermeiren E. (2012). Syndecan-syntenin-ALIX regulates the biogenesis of exosomes. Nat. Cell Biol..

[52] Bhattacharya S., Pal K., Sharma A.K., Dutta S.K., Lau J.S., Yan I.K., Wang E., Elkhanany A., Alkharfy K.M., Sanyal A. (2014). GAIP interacting protein c-terminus regulates autophagy and exosome biogenesis of pancreatic cancer through metabolic pathways. PLoS ONE.

[53] Mobius W., Ohno-Iwashita Y., van Donselaar E.G., Oorschot V.M., Shimada Y., Fujimoto T., Heijnen H.F., Geuze H.J., Slot J.W. (2002). Immunoelectron microscopic localization of cholesterol using biotinylated and non-cytolytic perfringolysin O. J. Histochem. Cytochem..

[54] Hemler M.E. (2005). Tetraspanin functions and associated microdomains. Nat. Rev. Mol. Cell Biol..

[55] Hemler M.E. (2008). Targeting of tetraspanin proteins—Potential benefits and strategies. Nat. Rev. Drug Discov..

[56] Yue S., Mu W., Erb U., Zöller M. (2015). The tetraspanins CD151 and TSPAN8 are essential exosome components for the crosstalk between cancer initiating cells and their surrounding. Oncotarget.

[57] De Jong O.G., Verhaar M.C., Chen Y., Vader P., Gremmels H., Posthuma G., Schiffelers R.M., Gucek M., van Balkom B.W. (2012). Cellular stress conditions are reflected in the protein and RNA content of endothelial cell-derived exosomes. J. Extracell. Vesicles.

[58] Milane L., Singh A., Mattheolabakis G., Suresh M., Amiji M.M. (2015). Exosome mediated communication within the tumor microenvironment. J. Control. Release.

[59] Kohlhapp F.J., Mitra A.K., Lengyel E., Peter M.E. (2015). MicroRNAs as mediators and communicators between cancer cells and the tumor microenvironment. Oncogene.

[60] Su Z., Yang Z., Xu Y., Chen Y., Yu Q. (2015). MicroRNAs in apoptosis, autophagy and necroptosis. Oncotarget.

[61] Penfornis P., Vallabhaneni K.C., Whitt J., Pochampally R. (2016). Extracellular vesicles as carriers of microRNA, proteins and lipids in tumor microenvironment. Int. J. Cancer.

[62] He L., Hannon G.J. (2004). MicroRNAs: Small RNAs with a big role in gene regulation. Nat. Rev. Genet..

[63] Dalmay T. (2013). Mechanism of miRNA-mediated repression of mRNA translation. Essays Biochem..

[64] Bartel D.P. (2009). MicroRNAs: Target recognition and regulatory functions. Cell.

[65] Zaravinos A. (2015). The regulatory role of microRNAs in EMT and cancer. J. Oncol..

[66] Raza U., Zhang J.D., Sahin O. (2014). MicroRNAs: Master regulators of drug resistance, stemness, and metastasis. J. Mol. Med..

[67] Guduric-Fuchs J., O’Connor A., Camp B., O’Neill C.L., Medina R.J., Simpson D.A. (2012). Selective extracellular vesicle-mediated export of an overlapping set of microRNAs from multiple cell types. BMC Genom..

[68] Gibbings D.J., Ciaudo C., Erhardt M., Voinnet O. (2009). Multivesicular bodies associate with components of miRNA effector complexes and modulate miRNA activity. Nat. Cell Biol..

[69] Squadrito M.L., Baer C., Burdet F., Maderna C., Gilfillan G.D., Lyle R., Ibberson M., de Palma M. (2014). Endogenous RNAs modulate microRNA sorting to exosomes and transfer to acceptor cells. Cell Rep..

[70] Villarroya-Beltri C., Gutierrez-Vazquez C., Sanchez-Cabo F., Perez-Hernandez D., Vazquez J., Martin-Cofreces N., Martinez-Herrera D.J., Pascual-Montano A., Mittelbrunn M., Sanchez-Madrid F. (2013). Sumoylated HNRNPA2B1 controls the sorting of miRNAs into exosomes through binding to specific motifs. Nat. Commun..

[71] Bolukbasi M.F., Mizrak A., Ozdener G.B., Madlener S., Strobel T., Erkan E.P., Fan J.B., Breakefield X.O., Saydam O. (2012). MiR-1289 and “zipcode”-like sequence enrich mrnas in microvesicles. Mol. Ther. Nucleic Acids.

[72] Koppers-Lalic D., Hackenberg M., Bijnsdorp I.V., van Eijndhoven M.A., Sadek P., Sie D., Zini N., Middeldorp J.M., Ylstra B., de Menezes R.X. (2014). Nontemplated nucleotide additions distinguish the small RNA composition in cells from exosomes. Cell Rep..

[73] Chevillet J.R., Kang Q., Ruf I.K., Briggs H.A., Vojtech L.N., Hughes S.M., Cheng H.H., Arroyo J.D., Meredith E.K., Gallichotte E.N. (2014). Quantitative and stoichiometric analysis of the microRNA content of exosomes. Proc. Natl. Acad. Sci. USA.

[74] Batagov A.O., Kurochkin I.V. (2013). Exosomes secreted by human cells transport largely mRNA fragments that are enriched in the 3′-untranslated regions. Biol. Direct..

[75] Bobrie A., Colombo M., Raposo G., Thery C. (2011). Exosome secretion: Molecular mechanisms and roles in immune responses. Traffic.

[76] Fevrier B., Raposo G. (2004). Exosomes: Endosomal-derived vesicles shipping extracellular messages. Curr. Opin. Cell Biol..

[77] Savina A., Fader C.M., Damiani M.T., Colombo M.I. (2005). Rab11 promotes docking and fusion of multivesicular bodies in a calcium-dependent manner. Traffic.

[78] Riches A., Campbell E., Borger E., Powis S. (2014). Regulation of exosome release from mammary epithelial and breast cancer cells—A new regulatory pathway. Eur. J. Cancer.

[79] Anastasiadou E., Slack F.J. (2014). Cancer. Malicious exosomes. Science.

[80] Mulcahy L.A., Pink R.C., Carter D.R. (2014). Routes and mechanisms of extracellular vesicle uptake. J. Extracell. Vesicles.

[81] McKelvey K.J., Powell K.L., Ashton A.W., Morris J.M., McCracken S.A. (2015). Exosomes: Mechanisms of uptake. J. Circ. Biomark..

[82] Chernomordik L.V., Kozlov M.M. (2008). Mechanics of membrane fusion. Nat. Struct. Mol. Biol..

[83] Jahn R., Lang T., Sudhof T.C. (2003). Membrane fusion. Cell.

[84] Jahn R., Sudhof T.C. (1999). Membrane fusion and exocytosis. Annu. Rev. Biochem..

[85] Parolini I., Federici C., Raggi C., Lugini L., Palleschi S., de Milito A., Coscia C., Iessi E., Logozzi M., Molinari A. (2009). Microenvironmental pH is a key factor for exosome traffic in tumor cells. J. Biol. Chem..

[86] Rabinovitch M. (1995). Professional and non-professional phagocytes: An introduction. Trends Cell Biol..

[87] Feng D., Zhao W.L., Ye Y.Y., Bai X.C., Liu R.Q., Chang L.F., Zhou Q., Sui S.F. (2010). Cellular internalization of exosomes occurs through phagocytosis. Traffic.

[88] Sahay G., Alakhova D.Y., Kabanov A.V. (2010). Endocytosis of nanomedicines. J. Control. Release.

[89] Grimmer S., van Deurs B., Sandvig K. (2002). Membrane ruffling and macropinocytosis in A431 cells require cholesterol. J. Cell Sci..

[90] Svensson K.J., Christianson H.C., Wittrup A., Bourseau-Guilmain E., Lindqvist E., Svensson L.M., Morgelin M., Belting M. (2013). Exosome uptake depends on ERK1/2-heat shock protein 27 signaling and lipid Raft-mediated endocytosis negatively regulated by caveolin-1. J. Biol. Chem..

[91] Ramachandran R., Pucadyil T.J., Liu Y.W., Acharya S., Leonard M., Lukiyanchuk V., Schmid S.L. (2009). Membrane insertion of the pleckstrin homology domain variable loop 1 is critical for dynamin-catalyzed vesicle scission. Mol. Biol. Cell.

[92] Fitzner D., Schnaars M., van Rossum D., Krishnamoorthy G., Dibaj P., Bakhti M., Regen T., Hanisch U.K., Simons M. (2011). Selective transfer of exosomes from oligodendrocytes to microglia by macropinocytosis. J. Cell Sci..

[93] Koumangoye R.B., Sakwe A.M., Goodwin J.S., Patel T., Ochieng J. (2011). Detachment of breast tumor cells induces rapid secretion of exosomes which subsequently mediate cellular adhesion and spreading. PLoS ONE.

[94] Nazarenko I., Rana S., Baumann A., McAlear J., Hellwig A., Trendelenburg M., Lochnit G., Preissner K.T., Zoller M. (2010). Cell surface tetraspanin TSPAN8 contributes to molecular pathways of exosome-induced endothelial cell activation. Cancer Res..

[95] Christianson H.C., Svensson K.J., van Kuppevelt T.H., Li J.P., Belting M. (2013). Cancer cell exosomes depend on cell-surface heparan sulfate proteoglycans for their internalization and functional activity. Proc. Natl. Acad. Sci. USA.

[96] Franzen C.A., Simms P.E., van Huis A.F., Foreman K.E., Kuo P.C., Gupta G.N. (2014). Characterization of uptake and internalization of exosomes by bladder cancer cells. BioMed Res. Int..

[97] Naslund T.I., Paquin-Proulx D., Paredes P.T., Vallhov H., Sandberg J.K., Gabrielsson S. (2014). Exosomes from breast milk inhibit HIV-1 infection of dendritic cells and subsequent viral transfer to CD4+ T cells. AIDS.

[98] Hao S., Bai O., Li F., Yuan J., Laferte S., Xiang J. (2007). Mature dendritic cells pulsed with exosomes stimulate efficient cytotoxic T-lymphocyte responses and antitumour immunity. Immunology.

[99] Record M., Subra C., Silvente-Poirot S., Poirot M. (2011). Exosomes as intercellular signalosomes and pharmacological effectors. Biochem. Pharmacol..

[100] Kanada M., Bachmann M.H., Contag C.H. (2016). Signaling by extracellular vesicles advances cancer hallmarks. Trends Cancer.

[101] Ji H., Greening D.W., Barnes T.W., Lim J.W., Tauro B.J., Rai A., Xu R., Adda C., Mathivanan S., Zhao W. (2013). Proteome profiling of exosomes derived from human primary and metastatic colorectal cancer cells reveal differential expression of key metastatic factors and signal transduction components. Proteomics.

[102] Kruger S., Abd Elmageed Z.Y., Hawke D.H., Worner P.M., Jansen D.A., Abdel-Mageed A.B., Alt E.U., Izadpanah R. (2014). Molecular characterization of exosome-like vesicles from breast cancer cells. BMC Cancer.

[103] Harris D.A., Patel S.H., Gucek M., Hendrix A., Westbroek W., Taraska J.W. (2015). Exosomes released from breast cancer carcinomas stimulate cell movement. PLoS ONE.

[104] Chauhan S., Danielson S., Clements V., Edwards N.J., Ostrand-Rosenberg S., Fenselau C. (2017). Surface glycoproteins of exosomes shed by myeloid-derived suppressor cells contribute to function. J. Proteome Res..

[105] Hakulinen J., Sankkila L., Sugiyama N., Lehti K., Keski-Oja J. (2008). Secretion of active membrane type 1 matrix metalloproteinase (MMP-14) into extracellular space in microvesicular exosomes. J. Cell. Biochem..

[106] Ung T.H., Madsen H.J., Hellwinkel J.E., Lencioni A.M., Graner M.W. (2014). Exosome proteomics reveals transcriptional regulator proteins with potential to mediate downstream pathways. Cancer Sci..

[107] Gajos-Michniewicz A., Duechler M., Czyz M. (2014). miRNA in melanoma-derived exosomes. Cancer Lett..

[108] Valadi H., Ekstrom K., Bossios A., Sjostrand M., Lee J.J., Lotvall J.O. (2007). Exosome-mediated transfer of mRNAs and microRNAs is a novel mechanism of genetic exchange between cells. Nat. Cell Biol..

[109] Pegtel D.M. (2013). Oncogenic herpesviruses sending mixed signals. Proc. Natl. Acad. Sci. USA.

[110] Lasser C., Alikhani V.S., Ekstrom K., Eldh M., Paredes P.T., Bossios A., Sjostrand M., Gabrielsson S., Lotvall J., Valadi H. (2011). Human saliva, plasma and breast milk exosomes contain RNA: Uptake by macrophages. J. Transl. Med..

[111] Dhondt B., Rousseau Q., de Wever O., Hendrix A. (2016). Function of extracellular vesicle-associated miRNAs in metastasis. Cell Tissue Res..

[112] Azmi A.S., Bao B., Sarkar F.H. (2013). Exosomes in cancer development, metastasis, and drug resistance: A comprehensive review. Cancer Metastasis Rev..

[113] Koga Y., Yasunaga M., Moriya Y., Akasu T., Fujita S., Yamamoto S., Matsumura Y. (2011). Exosome can prevent RNase from degrading microRNA in feces. J. Gastrointest. Oncol..

[114] Lombardo D., Siret C., Beloribi-Djefaflia S. (2015). Exosomal lipids impact on tumoral cell behavior. Cell Cycle.

[115] Ferguson S.W., Nguyen J. (2016). Exosomes as therapeutics: The implications of molecular composition and exosomal heterogeneity. J. Control. Release.

[116] Villarroya-Beltri C., Baixauli F., Gutierrez-Vazquez C., Sanchez-Madrid F., Mittelbrunn M. (2014). Sorting it out: Regulation of exosome loading. Semin. Cancer Biol..

[117] Roma-Rodrigues C., Pereira F., Alves de Matos A., Fernandes M., Baptista P.V., Fernandes A.R. Smuggling gold nanoparticles across cell types—A new role for exosomes in gene silencing.

[118] Muralidharan-Chari V., Clancy J.W., Sedgwick A., D’Souza-Schorey C. (2010). Microvesicles: Mediators of extracellular communication during cancer progression. J. Cell Sci..

[119] Zhang H.G., Grizzle W.E. (2014). Exosomes: A novel pathway of local and distant intercellular communication that facilitates the growth and metastasis of neoplastic lesions. Am. J. Pathol..

[120] Harp D., Driss A., Mehrabi S., Chowdhury I., Xu W., Liu D., Garcia-Barrio M., Taylor R.N., Gold B., Jefferson S. (2016). Exosomes derived from endometriotic stromal cells have enhanced angiogenic effects in vitro. Cell Tissue Res..

[121] Syn N., Wang L., Sethi G., Thiery J.P., Goh B.C. (2016). Exosome-mediated metastasis: From epithelial-mesenchymal transition to escape from immunosurveillance. Trends Pharmacol. Sci..

[122] Kalluri R., Weinberg R.A. (2009). The basics of epithelial-mesenchymal transition. J. Clin. Investig..

[123] Lin C.W., Kao S.H., Yang P.C. (2014). The miRNAs and epithelial-mesenchymal transition in cancers. Curr. Pharm. Des..

[124] Zhao L., Liu W., Xiao J., Cao B. (2015). The role of exosomes and “exosomal shuttle microRNA” in tumorigenesis and drug resistance. Cancer Lett..

[125] Bang C., Thum T. (2012). Exosomes: New players in cell-cell communication. Int. J. Biochem. Cell Biol..

[126] Shedden K., Xie X.T., Chandaroy P., Chang Y.T., Rosania G.R. (2003). Expulsion of small molecules in vesicles shed by cancer cells: Association with gene expression and chemosensitivity profiles. Cancer Res..

[127] Corcoran C., Rani S., O’Brien K., O’Neill A., Prencipe M., Sheikh R., Webb G., McDermott R., Watson W., Crown J. (2012). Docetaxel-resistance in prostate cancer: Evaluating associated phenotypic changes and potential for resistance transfer via exosomes. PLoS ONE.

[128] Lv M.M., Zhu X.Y., Chen W.X., Zhong S.L., Hu Q., Ma T.F., Zhang J., Chen L., Tang J.H., Zhao J.H. (2014). Exosomes mediate drug resistance transfer in MCF-7 breast cancer cells and a probable mechanism is delivery of P-glycoprotein. Tumour Biol..

[129] Taylor D.D., Gercel-Taylor C. (2008). MicroRNA signatures of tumor-derived exosomes as diagnostic biomarkers of ovarian cancer. Gynecol. Oncol..

[130] Rabinowits G., Gercel-Taylor C., Day J.M., Taylor D.D., Kloecker G.H. (2009). Exosomal microRNA: A diagnostic marker for lung cancer. Clin. Lung Cancer.

[131] Silva J., Garcia V., Rodriguez M., Compte M., Cisneros E., Veguillas P., Garcia J.M., Dominguez G., Campos-Martin Y., Cuevas J. (2012). Analysis of exosome release and its prognostic value in human colorectal cancer. Genes Chromosome Cancer.

[132] Duijvesz D., Versluis C.Y., van der Fels C.A., Vredenbregt-van den Berg M.S., Leivo J., Peltola M.T., Bangma C.H., Pettersson K.S., Jenster G. (2015). Immuno-based detection of extracellular vesicles in urine as diagnostic marker for prostate cancer. Int. J. Cancer.

[133] Meng X., Muller V., Milde-Langosch K., Trillsch F., Pantel K., Schwarzenbach H. (2016). Diagnostic and prognostic relevance of circulating exosomal miR-373, miR-200a, miR-200b and miR-200c in patients with epithelial ovarian cancer. Oncotarget.

[134] Vaksman O., Trope C., Davidson B., Reich R. (2014). Exosome-derived miRNAs and ovarian carcinoma progression. Carcinogenesis.

[135] Nilsson J., Skog J., Nordstrand A., Baranov V., Mincheva-Nilsson L., Breakefield X.O., Widmark A. (2009). Prostate cancer-derived urine exosomes: A novel approach to biomarkers for prostate cancer. Br. J. Cancer.

[136] Zhang W., Ni M., Su Y., Wang H., Zhu S., Zhao A., Li G. (2016). MicroRNAs in serum exosomes as potential biomarkers in clear-cell renal cell carcinoma. Eur. Urol. Focus.

[137] Eichelser C., Stuckrath I., Muller V., Milde-Langosch K., Wikman H., Pantel K., Schwarzenbach H. (2014). Increased serum levels of circulating exosomal microRNA-373 in receptor-negative breast cancer patients. Oncotarget.

[138] Yeh Y.Y., Ozer H.G., Lehman A.M., Maddocks K., Yu L., Johnson A.J., Byrd J.C. (2015). Characterization of CLL exosomes reveals a distinct microRNA signature and enhanced secretion by activation of BCR signaling. Blood.

[139] Chalmin F., Ladoire S., Mignot G., Vincent J., Bruchard M., Remy-Martin J.P., Boireau W., Rouleau A., Simon B., Lanneau D. (2010). Membrane-associated HSP72 from tumor-derived exosomes mediates STAT3-dependent immunosuppressive function of mouse and human myeloid-derived suppressor cells. J. Clin. Investig..

[140] Phuyal S., Hessvik N.P., Skotland T., Sandvig K., Llorente A. (2014). Regulation of exosome release by glycosphingolipids and flotillins. FEBS J..

[141] Peinado H., Aleckovic M., Lavotshkin S., Matei I., Costa-Silva B., Moreno-Bueno G., Hergueta-Redondo M., Williams C., Garcia-Santos G., Ghajar C. (2012). Melanoma exosomes educate bone marrow progenitor cells toward a pro-metastatic phenotype through MET. Nat. Med..

[142] Ostrowski M., Carmo N.B., Krumeich S., Fanget I., Raposo G., Savina A., Moita C.F., Schauer K., Hume A.N., Freitas R.P. (2010). Rab27a and Rab27b control different steps of the exosome secretion pathway. Nat. Cell Biol..

[143] Sung B.H., Ketova T., Hoshino D., Zijlstra A., Weaver A.M. (2015). Directional cell movement through tissues is controlled by exosome secretion. Nat. Commun..

[144] El Andaloussi S., Mager I., Breakefield X.O., Wood M.J. (2013). Extracellular vesicles: Biology and emerging therapeutic opportunities. Nat. Rev. Drug Discov..

[145] Tian Y., Li S., Song J., Ji T., Zhu M., Anderson G.J., Wei J., Nie G. (2014). A doxorubicin delivery platform using engineered natural membrane vesicle exosomes for targeted tumor therapy. Biomaterials.

[146] Vader P., Breakefield X.O., Wood M.J. (2014). Extracellular vesicles: Emerging targets for cancer therapy. Trends Mol. Med..

[147] Zhang H.G., Grizzle W.E. (2011). Exosomes and cancer: A newly described pathway of immune suppression. Clin. Cancer Res..

[148] Escudier B., Dorval T., Chaput N., Andre F., Caby M.P., Novault S., Flament C., Leboulaire C., Borg C., Amigorena S. (2005). Vaccination of metastatic melanoma patients with autologous dendritic cell (DC) derived-exosomes: Results of thefirst phase I clinical trial. J. Transl. Med..

[149] Morse M.A., Garst J., Osada T., Khan S., Hobeika A., Clay T.M., Valente N., Shreeniwas R., Sutton M.A., Delcayre A. (2005). A phase I study of dexosome immunotherapy in patients with advanced non-small cell lung cancer. J. Transl. Med..

[150] Dai S., Wei D., Wu Z., Zhou X., Wei X., Huang H., Li G. (2008). Phase I clinical trial of autologous ascites-derived exosomes combined with GM-CSF for colorectal cancer. Mol. Ther..

[151] Viaud S., Terme M., Flament C., Taieb J., Andre F., Novault S., Escudier B., Robert C., Caillat-Zucman S., Tursz T. (2009). Dendritic cell-derived exosomes promote natural killer cell activation and proliferation: A role for NKG2D ligands and IL-15Rα. PLoS ONE.

[152] ClinicalTrials.gov. https://clinicaltrials.gov/.

[153] Cabral R.M., Baptista P.V. (2014). Anti-cancer precision theranostics: A focus on multifunctional gold nanoparticles. Expert Rev. Mol. Diagn..

[154] Cabral R.M., Baptista P.V. (2013). The chemistry and biology of gold nanoparticle-mediated photothermal therapy: Promises and challenges. Nano LIFE.

[155] Link S., El-Sayed M.A. (2003). Optical properties and ultrafast dynamics of metallic nanocrystals. Annu. Rev. Phys. Chem..

[156] Dreaden E.C., Mackey M.A., Huang X., Kang B., El-Sayed M.A. (2011). Beating cancer in multiple ways using nanogold. Chem. Soc. Rev..

[157] Liu Y., Shipton M.K., Ryan J., Kaufman E.D., Franzen S., Feldheim D.L. (2007). Synthesis, stability, and cellular internalization of gold nanoparticles containing mixed peptide-poly(ethylene glycol) monolayers. Anal. Chem..

[158] Sanz V., Conde J., Hernández Y., Baptista P.V., Ibarra M.R., de la Fuente J.M. (2012). Effect of PEG biofunctional spacers and TAT peptide on dsRNA loading on gold nanoparticles. J. Nanopart. Res..

[159] Conde J., Dias J.T., Grazu V., Moros M., Baptista P.V., de la Fuente J.M. (2014). Revisiting 30 years of biofunctionalization and surface chemistry of inorganic nanoparticles for nanomedicine. Front. Chem..

[160] Hurst S.J., Lytton-Jean A.K., Mirkin C.A. (2006). Maximizing DNA loading on a range of gold nanoparticle sizes. Anal. Chem..

[161] Rosa J., Conde J., de la Fuente J.M., Lima J.C., Baptista P.V. (2012). Gold-nanobeacons for real-time monitoring of RNA synthesis. Biosens. Bioelectron..

[162] Conde J., Rosa J., de la Fuente J.M., Baptista P.V. (2013). Gold-nanobeacons for simultaneous gene specific silencing and intracellular tracking of the silencing events. Biomaterials.

[163] Conde J., Larguinho M., Cordeiro A., Raposo L.R., Costa P.M., Santos S., Diniz M.S., Fernandes A.R., Baptista P.V. (2014). Gold-nanobeacons for gene therapy: Evaluation of genotoxicity, cell toxicity and proteome profiling analysis. Nanotoxicology.

[164] Sato K., Hosokawa K., Maeda M. (2003). Rapid aggregation of gold nanoparticles induced by non-cross-linking DNA hybridization. J. Am. Chem. Soc..

[165] Storhoff J.J., Lucas A.D., Garimella V., Bao Y.P., Muller U.R. (2004). Homogeneous detection of unamplified genomic DNA sequences based on colorimetric scatter of gold nanoparticle probes. Nat. Biotechnol..

[166] Baptista P., Doria G., Henriques D., Pereira E., Franco R. (2005). Colorimetric detection of eukaryotic gene expression with DNA-derivatized gold nanoparticles. J. Biotechnol..

[167] Thaxton C.S., Georganopoulou D.G., Mirkin C.A. (2006). Gold nanoparticle probes for the detection of nucleic acid targets. Clin. Chim. Acta.

[168] Zhang X., Servos M.R., Liu J. (2012). Surface science of DNA adsorption onto citrate-capped gold nanoparticles. Langmuir.

[169] Lu W., Xiong C., Zhang G., Huang Q., Zhang R., Zhang J.Z., Li C. (2009). Targeted photothermal ablation of murine melanomas with melanocyte-stimulating hormone analog-conjugated hollow gold nanospheres. Clin. Cancer Res..

[170] Choi C.H., Alabi C.A., Webster P., Davis M.E. (2010). Mechanism of active targeting in solid tumors with transferrin-containing gold nanoparticles. Proc. Natl. Acad. Sci. USA.

[171] Melancon M.P., Lu W., Yang Z., Zhang R., Cheng Z., Elliot A.M., Stafford J., Olson T., Zhang J.Z., Li C. (2008). In vitro and in vivo targeting of hollow gold nanoshells directed at epidermal growth factor receptor for photothermal ablation therapy. Mol. Cancer Ther..

[172] Sperling R.A., Parak W.J. (2010). Surface modification, functionalization and bioconjugation of colloidal inorganic nanoparticles. Philos. Trans. A Math. Phys. Eng. Sci..

[173] Stuffers S., Sem Wegner C., Stenmark H., Brech A. (2009). Multivesicular endosome biogenesis in the absence of ESCRTS. Traffic.

[174] Li F., Li P., Yang L., Tang B. (2012). Simple and sensitive fluorescence detection of the RNA endonuclease activity of mammalian argonaute2 protein based on an RNA molecular beacon. Chem. Commun..

[175] Stenmark H., Parton R.G., Steele-Mortimer O., Lutcke A., Gruenberg J., Zerial M. (1994). Inhibition of Rab5 GTPase activity stimulates membrane fusion in endocytosis. EMBO J..

[176] Giuseppe D.N., Bugatti A., Zendrini A., Mazzoldi E.L., Montanelli A., Caimi L., Rusnati M., Ricotta D., Bergese P. (2016). Merging colloidal nanoplasmonics and surface plasmon resonance spectroscopy for enhanced profiling of multiple myeloma-derived exosomes. Biosens. Bioelectron..

[177] Duraichelvan R., Srinivas B., Badilescu S., Ouellette R., Ghosh A., Packirisamy M. (2016). Exosomes detection by a label-free localized surface plasmonic resonance method. ECS Trans..

[178] Oliveira-Rodriguez M., Lopez-Cobo S., Reyburn H.T., Costa-Garcia A., Lopez-Martin S., Yanez-Mo M., Cernuda-Morollon E., Paschen A., Vales-Gomez M., Blanco-Lopez M.C. (2016). Development of a rapid lateral flow immunoassay test for detection of exosomes previously enriched from cell culture medium and body fluids. J. Extracell Vesicles.

[179] Im H., Shao H., Park Y.I., Peterson V.M., Castro C.M., Weissleder R., Lee H. (2014). Label-free detection and molecular profiling of exosomes with a nano-plasmonic sensor. Nat. Biotechnol..

[180] Angeloni N.L., McMahon K.M., Swaminathan S., Plebanek M.P., Osman I., Volpert O.V., Thaxton C.S. (2016). Pathways for modulating exosome lipids identified by high-density lipoprotein-like nanoparticle binding to scavenger receptor type B-1. Sci Rep..

[181] Plebanek M.P., Mutharasan R.K., Volpert O., Matov A., Gatlin J.C., Thaxton C.S. (2015). Nanoparticle targeting and cholesterol flux through scavenger receptor type B-1 inhibits cellular exosome uptake. Sci. Rep..

[182] Paramelle D., Nieves D., Brun B., Kraut R.S., Fernig D.G. (2015). Targeting cell membrane lipid rafts by stoichiometric functionalization of gold nanoparticles with a sphingolipid-binding domain peptide. Adv. Healthc. Mater..

[183] Cox D., Tseng C.C., Bjekic G., Greenberg S. (1999). A requirement for phosphatidylinositol 3-kinase in pseudopod extension. J. Biol. Chem..

[184] Araki N., Johnson M.T., Swanson J.A. (1996). A role for phosphoinositide 3-kinase in the completion of macropinocytosis and phagocytosis by macrophages. J. Cell Biol..

[185] Wang L.H., Rothberg K.G., Anderson R.G. (1993). Mis-assembly of clathrin lattices on endosomes reveals a regulatory switch for coated pit formation. J. Cell Biol..

[186] Escrevente C., Keller S., Altevogt P., Costa J. (2011). Interaction and uptake of exosomes by ovarian cancer cells. BMC Cancer.

[187] Montecalvo A., Larregina A.T., Shufesky W.J., Stolz D.B., Sullivan M.L., Karlsson J.M., Baty C.J., Gibson G.A., Erdos G., Wang Z. (2012). Mechanism of transfer of functional microRNAs between mouse dendritic cells via exosomes. Blood.

[188] Nanbo A., Kawanishi E., Yoshida R., Yoshiyama H. (2013). Exosomes derived from epstein-barr virus-infected cells are internalized via caveola-dependent endocytosis and promote phenotypic modulation in target cells. J. Virol..

[189] Nativo P., Prior I.A., Brust M. (2008). Uptake and intracellular fate of surface-modified gold nanoparticles. ACS Nano.

[190] Johnsen K.B., Gudbergsson J.M., Skov M.N., Pilgaard L., Moos T., Duroux M. (2014). A comprehensive overview of exosomes as drug delivery vehicles—Endogenous nanocarriers for targeted cancer therapy. Biochim. Biophys. Acta.

[191] Marchesano V., Hernandez Y., Salvenmoser W., Ambrosone A., Tino A., Hobmayer B., de la Fuente J.M., Tortiglione C. (2013). Imaging inward and outward trafficking of gold nanoparticles in whole animals. ACS Nano.

[192] Mosesson Y., Mills G.B., Yarden Y. (2008). Derailed endocytosis: An emerging feature of cancer. Nat. Rev. Cancer.

[193] Wei J.X., Lv L.H., Wan Y., Cao Y., Li G., Lin H., Zhou R., Shang C.Z., Cao J., He H. (2015). Vps4a functions as a tumor suppressor by regulating the secretion and uptake of exosomal microRNAs in human hepatoma cells. Hepatology.

[194] Kanamala M., Wilson W.R., Yang M., Palmer B.D., Wu Z. (2016). Mechanisms and biomaterials in pH-responsive tumour targeted drug delivery: A review. Biomaterials.

[195] Vlodavsky I., Friedmann Y., Elkin M., Aingorn H., Atzmon R., Ishai-Michaeli R., Bitan M., Pappo O., Peretz T., Michal I. (1999). Mammalian heparanase: Gene cloning, expression and function in tumor progression and metastasis. Nat. Med..

[196] Kelly T., Miao H.Q., Yang Y., Navarro E., Kussie P., Huang Y., MacLeod V., Casciano J., Joseph L., Zhan F. (2003). High heparanase activity in multiple myeloma is associated with elevated microvessel density. Cancer Res..

[197] Edovitsky E., Elkin M., Zcharia E., Peretz T., Vlodavsky I. (2004). Heparanase gene silencing, tumor invasiveness, angiogenesis, and metastasis. J. Natl. Cancer Inst..

[198] Ilan N., Elkin M., Vlodavsky I. (2006). Regulation, function and clinical significance of heparanase in cancer metastasis and angiogenesis. Int. J. Biochem. Cell Biol..

[199] Kharaziha P., Ceder S., Li Q., Panaretakis T. (2012). Tumor cell-derived exosomes: A message in a bottle. Biochim. Biophys. Acta.

[200] Hendrix A., de Wever O. (2013). Rab27 GTPases distribute extracellular nanomaps for invasive growth and metastasis: Implications for prognosis and treatment. Int. J. Mol. Sci..

[201] Wang J.S., Wang F.B., Zhang Q.G., Shen Z.Z., Shao Z.M. (2008). Enhanced expression of Rab27A gene by breast cancer cells promoting invasiveness and the metastasis potential by secretion of insulin-like growth factor-II. Mol. Cancer Res..

[202] May R.C., Machesky L.M. (2001). Phagocytosis and the actin cytoskeleton. J. Cell Sci..

[203] Lamaze C., Fujimoto L.M., Yin H.L., Schmid S.L. (1997). The actin cytoskeleton is required for receptor-mediated endocytosis in mammalian cells. J. Biol. Chem..

[204] Kerr M.C., Teasdale R.D. (2009). Defining macropinocytosis. Traffic.

[205] Stephens L., Ellson C., Hawkins P. (2002). Roles of PI3Ks in leukocyte chemotaxis and phagocytosis. Curr. Opin. Cell Biol..

[206] Doherty G.J., McMahon H.T. (2009). Mechanisms of endocytosis. Annu. Rev. Biochem..

[207] Grassart A., Cheng A.T., Hong S.H., Zhang F., Zenzer N., Feng Y., Briner D.M., Davis G.D., Malkov D., Drubin D.G. (2014). Actin and dynamin2 dynamics and interplay during clathrin-mediated endocytosis. J. Cell Biol..

[208] Gout I., Dhand R., Hiles I.D., Fry M.J., Panayotou G., Das P., Truong O., Totty N.F., Hsuan J., Booker G.W. (1993). The GTPase dynamin binds to and is activated by a subset of SH3 domains. Cell.

[209] Yamada H., Takeda T., Michiue H., Abe T., Takei K. (2016). Actin bundling by dynamin 2 and cortactin is implicated in cell migration by stabilizing filopodia in human non-small cell lung carcinoma cells. Int. J. Oncol..

[210] Canton I., Battaglia G. (2012). Endocytosis at the nanoscale. Chem. Soc. Rev..

[211] Anderson R.G. (1998). The caveolae membrane system. Annu. Rev. Biochem..

[212] Nabi I.R., Le P.U. (2003). Caveolae/raft-dependent endocytosis. J. Cell Biol..

[213] Pedrosa P., Vinhas R., Fernandes A.R., Baptista P.V. (2015). Gold nanotheranostics: Proof-of-concpet or clinical tool?. Nanomaterials.

